# Standard Intravenous Concentrations in German Neonatal Intensive Care: Results of a National Consensus and Cross-Sectional Survey

**DOI:** 10.3390/jcm15082921

**Published:** 2026-04-11

**Authors:** Lisa Wende, Almuth Kaune, Mark Schoberer, Thorsten Orlikowsky, Dirk Wackernagel, Julia Haering-Zahn, Felix Schöne, Daniela Bach, Bianka Rösner, Sebastian Schubert, Rangmar Goelz, Irene Krämer, Karen B. Kreutzer, Albrecht Eisert

**Affiliations:** 1 Hospital Pharmacy, RWTH Aachen University Hospital, 52074 Aachen, Germany; lwende@ukaachen.de; 2 Pharmacy Department, University Hospital Carl Gustav Carus (AöR), TU Dresden, 01307 Dresden, Germany; 3 Section of Neonatology, Department of Pediatric and Adolescent Medicine, RWTH Aachen University Hospital, 52074 Aachen, Germany; 4 Department of Neonatology, Medical Center at the Johannes Gutenberg University Mainz, 55131 Mainz, Germany; 5 Department of Pediatrics and Adolescent Medicine, Universitätsklinikum Erlangen, Friedrich-Alexander-Universität Erlangen-Nürnberg (FAU), 91054 Erlangen, Germany; 6 Hospital Pharmacy, University Hospital Halle (Saale), 06120 Halle (Saale), Germany; felix.schoene@uk-halle.de; 7 Hospital Pharmacy, University Children’s Hospital Tübingen, 72076 Tübingen, Germany; 8 Department of Neonatology, Charité—Universitätsmedizin Berlin, 10117 Berlin, Germany; 9 Hospital Pharmacy, Medical Center at the Johannes Gutenberg University Mainz, 55131 Mainz, Germany; 10 Department of Neonatology, University Children’s Hospital Tübingen, 72076 Tübingen, Germanykaren.kreutzer@med.uni-tuebingen.de (K.B.K.); 11 Pharmacy Department, Johannes Gutenberg University Mainz, 55131 Mainz, Germany; 12 Institute of Clinical Pharmacology, RWTH Aachen University Hospital, 52074 Aachen, Germany

**Keywords:** neonatology, medication safety, patient safety, injections, intravenous, standardization

## Abstract

**Background/Objectives:** Medication errors remain a patient safety concern in neonatal intensive care units (NICU), mainly due to multiple dilution steps, a lack of standardized preparation instructions, and the frequent use of high-alert medications. While standard concentrations (SCs) for intravenous (iv) medication are recommended internationally, a national standard is missing for NICUs in Germany. The aim of this study was to evaluate a proposal for a national list of standardized iv medication concentrations to be used in German NICUs. **Methods**: In collaboration with the German Society for Neonatology and Pediatric Intensive Care (GNPI) and the Federal Association of German Hospital Pharmacists (ADKA), a multiprofessional expert team, including experts from the medication safety initiatives TELE-KASPER and Kinderformularium.DE and affiliated with seven German university hospitals, evaluated SCs for infusion medication administered to infants weighing 500 g to 5 kg. The evaluation process was based on international SCs lists, clinical practice, stability data, and handling aspects. Medication used in at least four of the seven hospitals was shortlisted. In the first round of the consensus process, an online survey submitted to the German Level-1 NICUs (n = 165) and their affiliated hospital pharmacies identified preferred SCs. In the second round of the consensus process, the expert team further evaluated the results of the survey. **Results**: The survey response rate was 52%. The consensus process resulted in a list encompassing 50 iv medications and 80 appropriate SCs. Ancillary information on preparation, stability, osmolarity, pH, and practical administration was added. **Conclusions**: The proposed SCs for infusion medication used in NICUs have the potential to reduce medication errors, simplify electronic prescribing, and improve workflow efficiency. Implementation aligns with international patient safety initiatives to improve medication safety in pediatric patients.

## 1. Introduction

Medication errors related to intravenous (iv) infusion in neonatal intensive care units (NICU) remain a concern for patient safety [1,2,3]. Studies report medication error rates ranging from 4 to 35.1 per 1000 patient-days in NICUs [2]. A German study found that 1.5% of error opportunities resulted in errors during iv medication preparation and administration in NICU/pediatric ICU settings [4]. Weight-based dosing and multiple dilution steps increase the likelihood of medication errors during prescribing, preparation, and administration in this specific population. The errors can have particularly severe consequences, since the patient population is vulnerable and high-alert medications are often used [5]. Since the early 2000s, various international organizations, including the American Society of Health-System Pharmacists (ASHP), the Royal College of Paediatrics and Child Health (RCPCH), and the Neonatal and Paediatric Pharmacy Group (NPPG), have recommended the use of standard concentrations (SCs) for pediatric and neonatal patients to minimize the risk of medication errors [6,7]. The established international literature, such as the Pediatric Injectable Drugs (The Teddy Bear Book), further supports standardized approaches to the preparation and administration of pediatric iv medications [8]. While a few countries, such as the UK, Ireland, Sweden, and Spain, have adopted SCs for iv medications in pediatric patients on a national level, it is more common for neonatal and pediatric units to develop SCs on their own [9,10,11,12,13]. In weight-based dosing, switching from variable concentrations with fixed infusion volumes to SCs with varying infusion volumes reduces the number of calculation steps and facilitates electronic prescribing [14]. Centralized preparation of infusion solutions in hospital pharmacies can increase the preparation accuracy and decrease the risk of antimicrobial contamination [15,16]. Studies implementing SCs accomplished positive outcomes, including reductions in prescribing and preparation errors, improvements in workflow efficiency, and increased staff satisfaction [14]. Moreover, clinical practice in NICUs is often based on individual approaches and local guidelines. SCs have been suggested as an approach to reduce variability and to provide a more evidence-based foundation for clinical decision-making [17].

Germany has established nationwide SCs for adult ICU patients but lacks a nationwide recommendation for neonatal SCs [18]. Most NICUs still depend on local SC lists or use varying concentrations that result from calculating doses based on weight. This variability can cause errors at care transition and requires a change in routines whenever health care staff move between institutions [19]. The aim of this study was to evaluate a national SC list to be used in German NICUs to improve medication and patient safety and to align with international patient safety initiatives [6,7,20,21]. The focus is on iv medication for neonatal patients weighing 500 g to 5 kg. Antineoplastic, electrolyte, and parenteral nutrition infusion solutions were not considered, as antineoplastic agents are rare in this population, and electrolytes and parenteral nutrition are multicomponent and individualized based on laboratory parameters.

## 2. Materials and Methods

Ethical approval (EK 25-259) was waived by the local ethics committee of the RWTH Aachen University Hospital, Aachen, Germany.

### 2.1. Project Initialization

The project was started in September 2023 by a multiprofessional expert team initially founded by two German university hospitals and subsequently expanded to seven university hospitals through the inclusion of experts from national medication safety initiatives (TELE-KASPER and Kinderformularium.DE) and professional societies (the German Society for Neonatology and Pediatric Intensive Care (GNPI) and the Federal Association of German Hospital Pharmacists (ADKA)). Members were ward pharmacists, pharmacists responsible for pharmacy-based preparation units, neonatologists, pediatricians, and a neonatal nursing expert. In one session, expert guests from the UK and Sweden shared their experiences and provided international input.

### 2.2. Compilation of a Medication Shortlist

In a pre-study, medication and SCs used in the NICUs of the seven founding university hospitals were compiled. In four online expert team meetings, the shortlist was evaluated based on the following aspects:

- Used in at least four of the seven NICUs;
- Recommended for neonates according to a positive benefit-risk ratio (criteria, e.g., adverse effects);
- Administered as bolus, short infusion, or continuous infusion;
- Licensed product available in Germany.

### 2.3. Evaluation of SCs to Be Listed

During eight online meetings, SCs of the medications on the shortlist to be used in preterm and term infants with a body weight ranging from 500 g to 5 kg, were evaluated. The consensus was made by assessing the following aspects using the experience of the expert team:

- SCs established at the NICUs of the expert team;
- SCs established in the USA, UK, and Sweden [6,7,22];
- Resulting volume load;
- Resulting infusion rate (minimum 0.1 mL per hour [23], maximum depending on the medication);
- Concentrations recommended in the summary of product characteristics (SmPCs) of licensed medicinal products;
- Physicochemical stability of SC infusion solution;
- Practical aspects of reconstitution/preparation (e.g., complexity of calculation, number of dilution steps, measurability of doses, volume expansion of drug solutions, concentrations of licensed medicinal products).

Volume expansion means that the volume of the reconstituted solution is slightly higher than the volume of diluent added to the vial. The actual concentration differs from the theoretical one, but should be used as a calculation basis. These medications require careful monitoring throughout the prescribing process, preparation, and calculation of infusion rates. Moreover, changes in the starting material may lead to different volume expansion and must be respected.

### 2.4. Nationwide Survey Regarding the SC Proposal List

Invitations to the nationwide survey were sent to the head physician of the German Level-1 NICUs (n = 165), listed in the IQTIG (German Institute for Quality Assurance and Transparency in Health Care) perinatal center registry (accessed December 2024), and the head of the affiliated pharmacy. The survey was conducted online (LimeSurvey GmbH, n.d., https://www.limesurvey.org/de, accessed between September 2024 and March 2025) and in a printed version between 19 January and 2 March 2025 (6 weeks). The survey access code was restricted to receive only one answer per NICU. After three weeks, reminder emails were sent to the participants, supplemented by a general announcement via the mailing list of the German Society of Hospital Pharmacists, ADKA e.V.

For each medication, participants were asked whether the proposed SC is appropriate in their setting. If not, respondents should report if the medication is not used at all, or which alternative concentration and administration route (bolus, short infusion, continuous infusion) should be listed as SC, and for what reason (see Figure S1).

### 2.5. Expert Evaluation of the SC Proposal List

In the second round of the consensus process, each expert initially reviewed the survey results to determine whether the proposed SCs should be kept or adjusted. This round was joined by two additional pediatricians. The results of the individual evaluation were compiled, and medications without full expert consensus on their concentrations were discussed. Final adjustments were made during four online meetings. To ensure appropriate infusion rates and volumes, the final SCs were cross-checked to match dosing recommendations of the German Kinderformularium.DE (accessed September 2025) [24].

### 2.6. Supplemental Information for the SC Proposal List

For each listed SC, practical instructions as well as published data on osmolarity, pH values, and stability were searched and added. Information sources included the SmPC, Kinderformularium.DE, Stabilis.org, and Päd i.v. database (see Table S1) [24,25,26]. In-use stability was evaluated for ready-to-administer solutions stored for 24 h at room temperature in polypropylene (PP) syringes. When compatibility data with PP containers was not available, information on other container materials was inserted. Limits set for peripheral vein administration were <900 mOsm/L and pH ≥ 5 and ≤9 [27,28,29]. When data of the proposed SC infusion solution were missing, data of similar concentrations or dilution conditions were given alternatively.

## 3. Results

**Figure 1:**
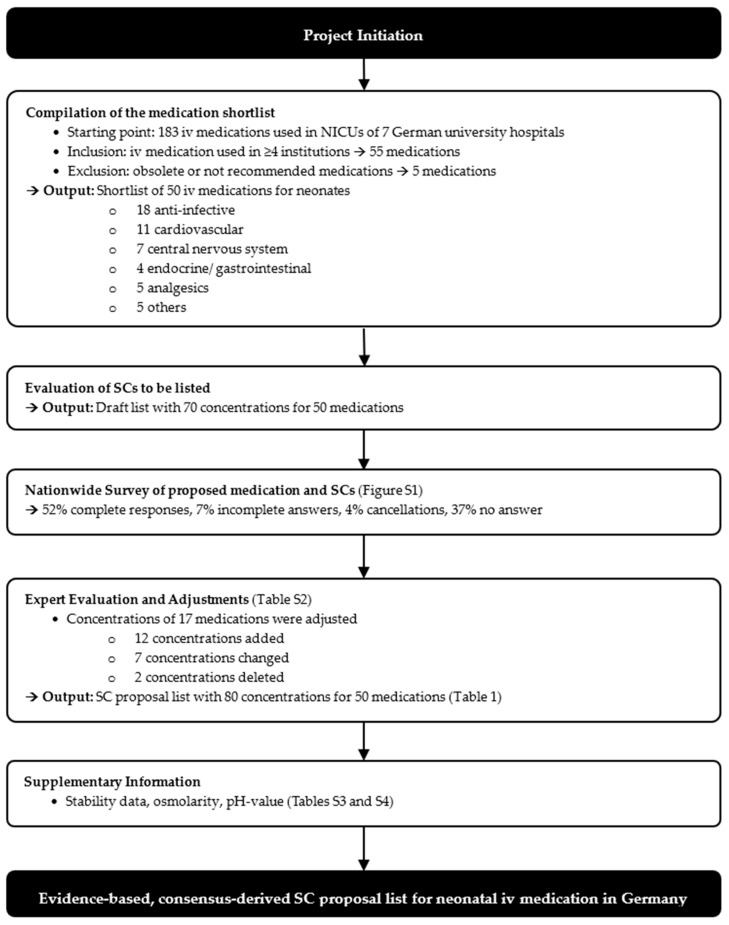
Flowchart of the development of a nationwide SC proposal list for neonatal iv medication. iv: intravenous, SC: standard concentration, NICU: neonatal intensive care.

Results of the evaluation process regarding iv medications and SCs to be used in neonatal patients (weight 500 g–5 kg) are shown in Figure 1.

The five medications excluded from the shortlist were amoxicillin/clavulanic acid, amphotericin B liposomal, metamizole, methylprednisolone, and piritramide, as a positive benefit–risk balance in neonatal use has not been demonstrated.

SCs were evaluated for 50 medications with a total of 70 proposed SCs in a nationwide survey among German NICUs. Table S2 provides a detailed summary of the responses, including the frequency of suggested alternative concentrations. Many alternative suggestions were weight-based, and overlaps of a specific alternative concentration across multiple sites were uncommon, which affects the interpretation of the number of alternative concentrations. Therefore, the evaluation of the proposed SCs could not rely on the ratio of approvals to suggested changes. Instead, it required expert judgment, which was carried out in the second consensus round, during which experts independently reviewed the survey results and assessed whether each SC should be kept or adjusted.

Medications for which full consensus was reached were retained unchanged, whereas those with partial agreement were discussed and adjusted as needed (see Table S2). For example, the SCs of aciclovir, alprostadil, and ampicillin were kept as proposed (100% agreement within the expert team), whereas the SC for amiodarone (5 mg/mL) was discussed again, as only 67% of experts agreed, and another SC of 1 mg/mL was added. The level of expert agreement on keeping the proposed SCs ranges from 17% to 100%. In total, the proposed SCs of 17 medications were changed. This approach of a second consensus round allowed the expert team to incorporate preferred alternative concentrations when appropriate and ensure that the final SCs reflected both the survey input and expert judgment.

The finalized German SC proposal list (see Table 1) includes 50 iv medications and 80 concentrations for neonates, including important information like off-label use, critical excipients, and deviating concentrations given in the SmPC.

Volume expansion during reconstitution is reported for ampicillin/sulbactam, ceftazidime, cefuroxime, and flucloxacillin. It is important to keep in mind that the actual concentrations after reconstitution differ from the theoretical concentrations based on the nominal diluent volume. In contrast to adult care, where the entire vial content is administered, volume expansion is of clinical relevance in neonatal and pediatric settings, where partial volumes are withdrawn from reconstituted vials. The SCs proposed in this list are based on actual concentrations. Due to product-specific volume expansion, deviations may occur in clinical practice. Therefore, cefuroxime should be reconstituted using the specified vial sizes, as different vial sizes may result in different actual concentrations. In neonatal and pediatric settings, caution is required when prescribing, preparing, and administering medications with volume expansion during reconstitution, as this variability may affect the accuracy of dosing. From a practical perspective, future efforts by manufacturers should aim to facilitate the preparation of SCs with round values (e.g., 50 or 100 mg/mL). Currently, achieving such concentrations often requires highly precise volume measurements with decimal places, which may be challenging in routine clinical practice. Therefore, the use of non-rounded SCs remains more feasible, as infusion rates typically require decimal precision anyway.

To achieve a peripherally applicable infusion, cefotaxime should only be reconstituted with sterile water for injection. Several medications contain critical excipients, including ethanol (e.g., alprostadil, phenobarbital) or propylene glycol (e.g., phenobarbital), which are listed to ensure consideration during prescribing. Supplementary information, such as preparation instructions, stability data, osmolarity, and pH values, is provided in Table S3. Data about feasible weight-adapted infusion rates and weight-based daily infusion volume in correlation to weight-based dosing rates are given in Table S4.

## 4. Discussion

The implementation of SCs for iv medication is an important strategy to reduce medication errors and enhance workflow efficiency in NICUs [14]. SCs enable closed-loop medication management with smart infusion pumps, reduce the need for bedside calculations, and harmonize preparation practices [34,35,36,37,38,39]. Consequently, the use of SCs can improve patient safety and consistency in clinical practice [14].

The scope of standardization and the degree of implementation and technological integration vary across countries [7,19,22,40,41]. Different from the other national frameworks, including neonatal and pediatric iv medication, this work focused on neonates with a body weight ranging from 500 g to 5 kg and iv medication administered as continuous infusion, short infusion, and bolus injection [7,19,22,41]. A nationwide SC list for pediatric patients > 5 kg body weight up to adults will be targeted in a follow-up project. The UK guideline focused on continuous infusions, whereas the Swedish guideline standardized concentrations for all administration routes (e.g., iv, oral, rectal, inhalation) [7,22,40]. Ireland has already developed a smart infusion pump database as a national standard of care [19,38]. Listing of clinically relevant information, such as off-label use and critical excipients, as well as preparation instructions, pH values, osmolarities, and in-use stability data, may be useful not only in Germany. Comparing the German SC proposal list with the SC lists published by ASHP, UK, and Sweden, the number of overlapping medications and SC is low (see Table 2) [6,7,22].

Eight medications (amiodarone, alprostadil, fentanyl, furosemide, heparin, human insulin, midazolam, and milrinone) are included in all four frameworks, but only two SCs are identical (1 IU/mL insulin and 1 mg/mL midazolam) [6,7,22]. Several factors may explain these discrepancies. First, the included patient populations differ between frameworks (neonatal versus pediatric populations), which leads to different dosing requirements and daily fluid volume ranges. While the German SC proposal list focuses on neonates weighing 500 g to 5 kg, the UK framework and the Swedish ePed include all children under 18 years of age, whereas the ASHP framework defines SCs for children weighing less than 50 kg [6,7,22]. Second, while most licensed medicinal products in Europe are available in similar vial sizes and concentrations, additional vial strengths (e.g., for many antibiotics and catecholamines) as well as different concentrations (e.g., heparin and phenobarbital) are available in the United States. National differences in licensed medicinal products and available vial sizes may influence feasible SCs. Third, variations in clinical practice, such as administration routes, fluid restrictions, and preparation protocols, may contribute to divergent SCs. Finally, regulatory requirements and national guidelines may further influence SC proposals.

The low degree of overlap may limit the transferability of safety strategies, such as smart infusion pump drug libraries, and increase the risk of medication errors in settings involving international staff or cross-border care. Therefore, future efforts should focus on international harmonization. Collaborative initiatives between international societies and regulatory authorities may support the development of consensus-based international SCs while considering national constraints.

The proposed SCs aim to provide as many concentrations as necessary, but as few as possible to minimize confusion and the risk of mix-ups [34,36]. Nevertheless, not all clinical scenarios can be fully covered by SCs. Patient-individual dosing or fluid volume needs may still require patient-specific preparation and concentrations [42]. As some proposed SCs are not in accordance with the SmPC, their use remains off-label and falls under the responsibility of the prescribing neonatologist. However, the development of SCs, based on clinical experience across multiple German NICUs and complemented by stability data, provides a scientific framework for decision support. This approach facilitates a shift from individual practices toward a more evidence-based use. There is still information about stability, compatibility with PP syringes, osmolarities, and pH values missing. Further studies are needed to investigate the physicochemical characteristics and ensure safe use of proposed SCs. We encourage manufacturers of licensed medicinal products to evaluate appropriate SCs, provide preparation instructions, and compatibility data with 50 mL syringes used as primary containers in the SmPC.

A safe and sustainable implementation of SCs requires multiprofessional collaboration among physicians, pharmacists, and nurses. Developing staff training strategies, labeling strategies, and integration into computerized physician order entry and smart infusion pump libraries are highly recommended procedures when introducing SCs [36,43]. In hospitals that already use institution-specific SCs, alignment with national standards should be approached cautiously and implemented gradually to ensure safety and acceptance. Ongoing revision will be necessary to adapt the proposed SCs to clinical feedback and emerging evidence. Establishing a centralized database for smart infusion pumps could ensure consistent use of SCs across hospitals [19].

## 5. Conclusions

The development of a national SC list for neonatal iv infusions, covering 50 medications with 80 concentrations, is an important step toward safe and efficient usage of neonatal medication in Germany. By reducing variability in prescribing, preparation, and administration, the proposed SCs assist patient safety and facilitate the integration of closed-loop medication management. However, implementation will require changes in established practices and acceptance in clinical settings. Continuous evaluation and future updates based on practical experience and emerging evidence will be necessary. In the future, national and international harmonization of SCs may further enhance patient safety in this vulnerable patient population.

## Figures and Tables

**Table 1:** German SC proposal list for neonatal intravenous medication.

Nr.	Medication	Recommended Standard Concentration	Administration Route	Ancillary Information
**1**	Aciclovir	5 mg/mL	short infusion	
**2**	Alprostadil	1 µg/mL	continuous infusion	**Contains ethanol!**
		2 µg/mL	continuous infusion	
**3**	Amiodarone HCl	1 mg/mL	continuous infusion	**Off-label** **Contains benzyl alcohol!** Central venous administration if continuous infusion or >2 mg/mL
		5 mg/mL	bolus	
**4**	Ampicillin	100 mg/mL	bolus	
**5**	Ampicillin/Sulbactam	100/50 mg/mL	bolus	**Volume expansion!** After adding 3.2 mL WFI, 4.2 mL can be withdrawn from the vial.
**6**	Cefotaxime	100 mg/mL	bolus/short infusion	Due to osmolarity, cefotaxime must only be dissolved with WFI.
**7**	Ceftazidime	90 mg/mL	bolus/short infusion	**Volume expansion!** Reconstitution results in a concentration of 90 mg/mL.
**8**	Cefuroxime	116 mg/mL	bolus	**Volume expansion!** Reconstitution results in a concentration of 116 mg/mL, using 250 mg and 750 mg vials.
**9**	Clarithromycin	2 mg/mL	short infusion	**Off-label**
**10**	Clindamycin	5 mg/mL	short infusion	**Off-label under 4 weeks!** Ratiopharm^®^ does not contain benzyl alcohol. Other licensed products do.
**11**	Clonidine HCl	1.5 µg/mL	continuous infusion	**Off-label**
		7.5 µg/mL	continuous infusion	
**12**	Caffeine citrate	20 mg/mL	bolus/short infusion	
**13**	Dexamethasone-21-dihydrogenphosphate	0.1 mg/mL	bolus	**Off-label**
		0.5 mg/mL	bolus	
**14**	Dobutamine	0.5 mg/mL	continuous infusion	**Off-label in preterm infants!** Central venous administration > 1 mg/mL
		1 mg/mL	continuous infusion	
		2.5 mg/mL	continuous infusion	
**15**	Dopamine HCl	0.5 mg/mL	continuous infusion	**Off-label** Central venous administration
		1 mg/mL	continuous infusion	
		2.5 mg/mL	continuous infusion	
**16**	Doxapram HCl · 1 H_2_O	1 mg/mL	continuous infusion	Must be administered as a push infusion.
		2 mg/mL	continuous infusion	
**17**	Epinephrine	10 µg/mL	continuous infusion	**Contains sodium metabisulfite!** (max. 0.5 mg/mL) Concentrations differ from SmPC.
		20 µg/mL	continuous infusion	
		40 µg/mL	continuous infusion	
**18**	Erythromycin	5 mg/mL	short infusion	**Off-label in preterm infants!**
**19**	Esketamine	0.5 mg/mL	bolus/continuous infusion	
		5 mg/mL	bolus/continuous infusion	
**20**	Esmolol HCl	10 mg/mL	continuous infusion	**Off-label**
**21**	Fentanyl	2.5 µg/mL	bolus/continuous infusion	**Off-label**
		5 µg/mL	bolus/continuous infusion	
		10 µg/mL	bolus/continuous infusion	
**22**	Flucloxacillin	48.5 mg/mL	short infusion	**Volume expansion!** Reconstitution results in a concentration of 48.5 mg/mL.
**23**	Fluconazole	2 mg/mL	short infusion	**Off-label in preterm infants!**
**24**	Furosemide	0.2 mg/mL	continuous infusion	**Off-label under 1 month!**
		0.4 mg/mL	continuous infusion	
		1 mg/mL	bolus/continuous infusion	
		5 mg/mL	bolus	
**25**	Gentamicin	2 mg/mL	short infusion	**Off-label in preterm infants!**
**26**	Heparin Na	1 IU/mL	continuous infusion	**Off-label under 1 month!**
		100 IU/mL	continuous infusion	
**27**	Human Insulin	0.02 IU/mL	continuous infusion	**Contains phenol!** **Beware of insulin adsorption to infusion lines and syringes:** Before administration, prime the line for 20 to 60 min with 20 mL 5 IU/mL insulin solution and flush with the insulin solution to be administered, afterwards. Monitor the first 3 to 6 h for overdosing [30,31,32,33]. Concentrations differ from SmPC.
		0.2 IU/mL	continuous infusion	
		1 IU/mL	continuous infusion	
**28**	Hydrocortisone	1 mg/mL	bolus	**Off-label**
**29**	Ibuprofen	5 mg/mL	short infusion	
**30**	Indomethacin	0.2 mg/mL	short infusion	Imported medicinal product
**31**	Levetiracetam	10 mg/mL	short infusion	**Off-label**
**32**	Meropenem	10 mg/mL	short infusion, also prolonged	**Off-label**
**33**	Metronidazole	5 mg/mL	short infusion	
**34**	Midazolam	0.1 mg/mL	bolus	Manual injection feasible. **Beware of neurotoxicity!**
		1 mg/mL	bolus	
**35**	Milrinone	50 µg/mL	continuous infusion	
		200 µg/mL	continuous infusion	
**36**	Morphine salts	10 µg/mL	continuous infusion	Morphine HCl and sulfate salts are treated equally regarding concentrations and dosing.
		100 µg/mL	bolus/continuous infusion	
		200 µg/mL	bolus/continuous infusion	
**37**	Naloxone HCl	40 µg/mL	bolus	
		100 µg/mL	bolus	
**38**	Norepinephrine	10 µg/mL	continuous infusion	**Off-label** Central venous administration is preferred.
		20 µg/mL	continuous infusion	
		40 µg/mL	continuous infusion	
**39**	Paracetamol	10 mg/mL	short infusion	**Off-label in preterm infants!** **Beware of overdosing!**
**40**	Penicillin G	0.1 Mio IU/mL	bolus	
**41**	Phenobarbital (base)	10 mg/mL	bolus/short infusion	**Contains ethanol and propylene glycol!** **CAUTION: Dosage and concentration are given as base, not as sodium salt.** Concentrations differ from SmPC.
**42**	Phytomenadione (Vitamin K1)	1 mg/mL	bolus	Concentrations differ from SmPC.
**43**	Piperacillin/Tazobactam	100/12.5 mg/mL	short infusion, also prolonged	**Off-label**
**44**	Prednisolone-21-hydrogensuccinate	1 mg/mL	bolus	
		5 mg/mL	bolus	
**45**	Propofol	5 mg/mL	bolus	**Off-label**
**46**	Sildenafil	0.16 mg/mL	continuous infusion	**Off-label**
		0.8 mg/mL	bolus/short infusion	
**47**	Teicoplanin	10 mg/mL	short infusion	
**48**	Theophylline	1 mg/mL	continuous infusion	**Off-label**
**49**	Vancomycin HCl	5 mg/mL	short infusion	
**50**	Vecuronium Br	0.1 mg/mL	bolus/continuous infusion	
		1 mg/mL	bolus/continuous infusion	

SmPC: summary of product characteristics, WFI: sterile water for injection.

**Table 2:** Comparison of the German SC proposal list to international SC frameworks.

	UK [7]	Sweden [22]	ASHP [6]	All Frameworks [6,7,22]
Overlapping medication [*n*]	16	27	17	8
Overlapping SC [*n*]	19	14	14	2

ASHP: American Society of Health-System Pharmacists, SC: standard concentration.

## Data Availability

The original contributions presented in this study are included in the article/Supplementary Material. Further inquiries can be directed to the corresponding author.

## Supplementary Materials

The following supporting information can be downloaded at: https://www.mdpi.com/article/10.3390/jcm15082921/s1, Table S1. Databases used for supporting information on SCs, Table S2. Survey results and expert evaluation of the SC proposal list, Table S3. Information on preparation and administration of the SC Proposal List, References–Table S3, Table S4. Information on Dosing, Infusion Rates, and Infusion Volumes of the SC proposal list, Figure S1. Nationwide survey on the SC proposal list (printed version; survey translated from German).

## Abbreviations

The following abbreviations are used in this manuscript:

ADKA	Federal Association of German Hospital Pharmacists
ASHP	American Society of Health-System Pharmacists
GNPI	German Society for Neonatology and Pediatric Intensive Care
IQTIG	German Institute for Quality Assurance and Transparency in Health Care
iv	intravenous
NICU	neonatal intensive care unit
NPPG	Neonatal and Paediatric Pharmacy Group
PP	polypropylene
RCPCH	Royal College of Paediatrics and Child Health
SC	standard concentration
SmPC	summary of product characteristic
WFI	sterile water for injection
