# Supplementary material for: Standard Intravenous Concentrations in German Neonatal Intensive Care: Results of a National Consensus and Cross-Sectional Survey
Source: J Clin Med. 2026 Apr 11;15(8):2921. doi: 10.3390/jcm15082921 (PMC13116128; doi:10.3390/jcm15082921)
Supplement: Supplementary file 1 [file jcm-15-02921-s001.zip › jcm-4225269-supplementary.pdf]

Table S1. Databases used for supporting information on SCs.

Database	Explanation	Importance for the Project
**Summary of product characteristics**	• Official document<br>• Information on formulation, indications, dosing, administration, contraindications, and stability information<br>• Legal and regulatory reference	• Provides approved dosing, preparation data and official stability data
**Kinderformularium.DE**	• German evidence-based pediatric medication database<br>• Offers dosing and type of administration recommendations, e.g. bolus or continuous infusion<br>• Based on literature and expert consensus	• Provides pediatric dosing ranges and off-label guidance
**Stabilis.org**	• International database on in-use stability of ready-to-administer infusion solutions<br>• Includes information on primary containers<br>• Referenced to scientific literature and official documents	• Provides evidence-based physicochemical stability data
**Päd i.v.**	• German national database (ADKA/DPhG)<br>• Focus on pediatric intravenous preparations and stability data<br>• Referenced to BNF for Children and Pediatric Injectable Drugs	• Provides stability data, osmolarity data and pH-values

Päd i.v.: Database for Pediatric Intravenous Drugs, ADKA: Federal Association of German Hospital Pharmacists, DPhG: German Pharmaceutical Society.

Table S2. Survey results and expert evaluation of the SC proposal list.

Medication	Answers	Count	Suggested Concentrations	Expert Evaluation (*n*=12)	Changes
Aciclovir	5 mg/mL	62	6x 2.5 mg/mL, 5x 10 mg/mL, 4x 25 mg/mL, 2x 2 mg/mL, 1x 4.17 mg/mL, 1x 50 mg/mL	Keep SC as it is. (100%)	No Changes.
	Not applicable	10			
	Alternative concentration	19			
	Not completed	11			
Alprostadil	1µg/mL	36	8x 5 µg/mL, 5x 10 µg/mL, 3x 1 µg/mL, 3x 3 µg/mL, 3x 6 µg/mL, 2x 0.5 µg/mL, 2x 1.5 µg/mL, 2x 4 µg/mL, 1x 0.25 µg/mL, 1x 2 µg/mL, 6x weight-based	Keep SC as it is. (100%)	No Changes.
	2 µg/mL	28			
	Not applicable	18			
	Alternative concentration	29			
	Not completed	11			
Amiodarone HCl	5 mg/mL	41	7x 50 mg/mL, 6x 2 mg/mL, 3x 3 mg/mL, 3x 1 mg/mL, 3x 6 mg/mL, 1x 10 mg/mL, 1x 5 mg/mL, 2x weight-based	Keep SC as it is. (67%)<br>Adjusting SC. (34%)	1 mg/mL added.
	Not applicable	25			
	Alternative concentration	23			
	Not completed	11			
Ampicillin	100 mg/mL	74	4x 50 mg/mL, 2x 100 mg/mL, 1x 30 mg/mL, 1x 20 mg/mL	Keep SC as it is. (100%)	No Changes.
	Not applicable	7			
	Alternative concentration	10			
	Not completed	11			
Ampicillin/ Sulbactam	250/125 mg/mL	33	15x 100/50 mg/mL, 15x 20/10 mg/mL, 2x 50/25 mg/mL, 6x 30/15 mg/mL, 2x 15 mg/mL, 2x 1000/500 mg/3 mL, 1x 10 mg/mL, 1x 5/2.5 mg/mL, 1x 200 mg/mL, 1x 12.5 mg/mL, 1x undiluted, 1x weight-based	Keep SC as it is. (33%)<br>Adjusting SC. (67%)	250/125 mg/mL changed to 100/50 mg/mL.
	Not applicable	10			
	Alternative concentration	46			
	Not completed	11			
Cefotaxime	50 mg/mL	48	35x 100 mg/mL, 2x 20 mg/mL, 2x 40 mg/mL, 1x 50 mg/mL, 1x 250 mg/mL	Keep SC as it is. (33%)<br>Adjusting SC. (67%)	50 mg/ mL changed to 100 mg/mL.
	Not applicable	5			
	Alternative concentration	37			
	Not completed	11			
Ceftazidime	30 mg/mL	26	15x 100 mg/mL, 6x 40 mg/mL, 4x 20 mg/mL, 1x 45 mg/mL, 1x 90 mg/mL, 1x 9 mg/mL, 1x 25 mg/mL, 1x 10 mg/mL, 1x weight-based	Keep SC as it is. (42%)<br>Adjusting SC. (58%)	30 mg/mL deleted.
	90 mg/mL	33			
	Not applicable	13			
	Alternative concentration	35			
	Not completed	11			
Cefuroxime	116 mg/mL	33	16x 100 mg/mL, 12x 50 mg/mL, 6x 30 mg/mL, 5x 75 mg/mL, 3x 15 mg/mL, 2x 46.4 mg/mL, 1x 125 mg/mL, 1x 5 mg/mL, 1x weight-based	Keep SC as it is. (100%)	No Changes.
	Not applicable	14			
	Alternative concentration	41			
	Not completed	11			
Clarithromycin	2 mg/mL	53	1x 50 mg/mL, 1x 25 mg/mL, 1x 5 mg/mL, 1x 0.5 mg/mL, 1x according to SmPC, 1x weight-based	Keep SC as it is. (100%)	No Changes.
	Not applicable	26			
	Alternative concentration	7			
	Not completed	11			
Clindamycin	5 mg/mL	28	15x 12 mg/mL, 9x 10 mg/mL, 5x 6 mg/mL, 3x 150 mg/mL, 2x 15 mg/mL, 1x 3 mg/mL, 1x 9 mg/mL, 1x weight-based	Keep SC as it is. (50%)<br>Adjusting SC. (50%)	No Changes.
	Not applicable	25			
	Alternative concentration	35			
	Not completed	11			
Clonidine HCl	1.5 µg/mL	30		Keep SC as it is. (100%)	No Changes.

Medication	Answers	Count	Suggested Concentrations	Expert Evaluation (*n*=12)	Changes
	7.5 µg/mL	23	11x 10 µg/mL, 9x 3 µg/mL, 8x 15 µg/mL, 7x 6 µg/mL, 5x 5 µg/mL, 5x 1 µg/mL, 3x 9 µg/mL, 2x 2 µg/mL, 2x 30 µg/mL, 2x 12 µg/mL, 1x 36 µg/mL, 1x 0.6 µg/mL, 1x 4 µg/mL, 1x 150 µg/mL, 1x 4.5 µg/mL, 1x 7.5 µg/mL, 6x weight-based		
	Not applicable	11			
	Alternative concentration	49			
	Not completed	11			
Caffeine citrate	20 mg/mL	64	9x 10 mg/mL, 5x 5 mg/mL, 2x 4 mg/mL, 2x 2 mg/mL, 3x weight-based	Keep SC as it is. (100%)	No Changes.
	Not applicable	5			
	Alternative concentration	19			
	Not completed	11			
Dexamethasone-21-dihydrogenphosphate	0.1 mg/mL	28	8x 4 mg/mL, 8x 1 mg/mL, 1x 0.2 mg/mL, 1x 0.05 mg/mL, 1x 8 mg/mL, 3x weight-based	Keep SC as it is. (92%) Adjusting SC. (8%)	No Changes.
	0.5 mg/mL	22			
	Not applicable	31			
	Alternative concentration	24			
	Not completed	11			
Dobutamine	1 mg/mL	43	13x 5 mg/mL, 8x 3 mg/mL, 6x 2 mg/mL, 5x 1.5 mg/mL, 4x 0.5 mg/mL, 3x 0.6 mg/mL,2x 2.5 mg/mL, 2x 0.75 mg/mL, 1x 0.2 mg/mL, 12x weight-based	Keep SC as it is. (50%) Adjusting SC. (50%)	0.5 mg/mL and 2.5 mg/mL added.
	Not applicable	10			
	Alternative concentration	43			
	Not completed	11			
Dopamine HCl	1 mg/mL	30	6x 5 mg/mL, 5x 2 mg/mL, 5x 1.5 mg/mL, 4x 0.5 mg/mL, 2x 0.75 mg/mL, 2x 3 mg/mL, 1x 2.5 mg/mL, 1x 1.2 mg/mL, 1x 2.4 mg/mL, 1x 0.6 mg/mL, 1x 80 mg/mL, 1x 0.2 mg/mL, 1x 10 mg/mL, 1x 0.1 mg/mL, 9x weight-based	Keep SC as it is. (50%) Adjusting SC. (50%)	0.5 mg/mL and 2.5 mg/mL added.
	Not applicable	33			
	Alternative concentration	32			
	Not completed	11			
Doxapram HCl · 1 H_2O	2.5 mg/mL	27	15x 1 mg/mL, 9x 2 mg/mL, 2x 5 mg/mL, 1x 0.5 mg/mL, 1x 20 mg/mL, 7x weight-based	Keep SC as it is. (42%) Adjusting SC. (58%)	2.5 mg/mL deleted. 1 mg/mL and 2 mg/mL added.
	Not applicable	30			
	Alternative concentration	34			
	Not completed	11			
Epinephrine	10 µg/mL	36	12x 20 µg/mL, 10x 100 µg/mL, 9x 30 µg/mL, 8x 60 µg/mL, 3x 6 µg/mL, 3x 5 µg/mL, 2x 15 µg/mL, 1x 25 µg/mL, 1x 50 µg/mL, 1x 200 µg/mL, 1x 1 mg/mL, 1x 4 µg/mL, 13x weight-based	Keep SC as it is. (58%) Adjusting SC. (42%)	20 µg/mL added.
	40 µg/mL	25			
	Not applicable	11			
	Alternative concentration	50			
	Not completed	11			
Erythromycin	5 mg/mL	44	5x 4 mg/mL, 4x 2 mg/mL, 4x 50 mg/mL, 3x 10 mg/mL, 1x 1 mg/mL, 1x 0.2 mg/mL, 1x 2.5 mg/mL	Keep SC as it is. (100%)	No Changes.
	Not applicable	26			
	Alternative concentration	19			
	Not completed	11			
Esketamine	0.5 mg/mL	29	5x 10 mg/mL, 4x 25 mg/mL, 4x 1 mg/mL, 2x 12.5 mg/mL, 1x 2 mg/mL, 6x weight-based	Keep SC as it is. (100%)	No Changes.
	5 mg/mL	51			
	Not applicable	16			
	Alternative concentration	21			
	Not completed	11			
Esmolol HCl	10 mg/mL	31	1x 1 mg/mL, 2x weight-based	Keep SC as it is. (100%)	No Changes.
	Not applicable	52			
	Alternative concentration	3			
	Not completed	11			
Fentanyl	2.5 µg/mL	18	6x 50 µg/mL, 3x 20 µg/mL, 1x 30 µg/mL, 1x 1 µg/mL, 1x 4 µg/mL, 10x weight-based	Keep SC as it is. (83%) Adjusting SC. (17%)	No Changes.
	5 µg/mL	31			
	10 µg/mL	44			
	Not applicable	19			
	Alternative concentration	21			
	Not completed	11			
Flucloxacillin	50 mg/mL	60	3x 10 mg/mL, 2x 20 mg/mL, 2x 40 mg/mL, 1x 5 mg/mL, 1x 100 mg/mL, 1x according to SmPC, 1x weight-based	Keep SC as it is. (92%) Adjusting SC. (8%)	50 mg/mL changed to 48.5 mg/mL, due to volume expansion.
	Not applicable	20			
	Alternative concentration	11			
	Not completed	11			
Fluconazole	2 mg/mL	78	1x 20 mg/mL, 1x 0.2 mg/mL	Keep SC as it is. (100%)	No Changes.
	Not applicable	8			
	Alternative concentration	2			
	Not completed	11			
Furosemide	0.2 mg/mL	53	16x 10 mg/mL, 3x 2 mg/mL, 2x 1.2 mg/mL, 1x 3 mg/mL, 1x 1.6 mg/mL, 6x weight-based	Keep SC as it is. (92%) Adjusting SC. (8%)	No Changes.
	0.4 mg/mL	23			
	1 mg/mL	32			
	5 mg/mL	22			
	Not applicable	11			
	Alternative concentration	29			
	Not completed	11			
Gentamicin	2 mg/mL	39	10x 1 mg/mL, 6x 5 mg/mL, 4x 10 mg/mL, 2x 0.8 mg/mL, 1x 4 mg/mL, 1x 4 mg/3mL, 1x 0.66 mg/mL, 1x 6.6 mg/mL, 1x 1.2 mg/mL, 1x 8 mg/mL, 1x 3 mg/mL, 4x weight-based	Keep SC as it is. (83%) Adjusting SC. (17%)	No Changes.
	Not applicable	18			
	Alternative concentration	32			
	Not completed	11			
Heparin Na	1 IU/mL	40	6x 10 IE/mL, 3x 0.5 IE/mL, 3x 20 IE/mL, 2x 50 IE/mL, 2x 200 IE/mL, 1x 1 IE/mL, 1x 2 IE/mL, 1x 100 IE/mL, 6x weight-based	Keep SC as it is. (83%) Adjusting SC. (17%)	No Changes.
	100 IU/mL	43			
	Not applicable	20			
	Alternative concentration	19			
	Not completed	11			
Human Insulin	0.002 IU/mL	16	8x 0.1 IE/mL, 4x 0.5 IE/mL, 3x 0.05 IE/mL, 2x 0.02 IE/mL, 2x 100 IE/mL, 1x 0.025 IE/mL, 1x	Keep SC as it is. (17%) Adjusting SC. (83%)	0.002 IU/mL changed to 0.02 IU/mL.
	0.2 IU/mL	32			
	1 IU/mL	32			

Medication	Answers	Count	Suggested Concentrations	Expert Evaluation (*n*=12)	Changes
	Not applicable	18	0.4 mg/mL, 1x 0.005 IE/mL, 13x weight-based		
	Alternative concentration	31			
	Not completed	11			
Hydrocortisone	1 mg/mL	56	9x 2 mg/mL, 4x 10 mg/mL, 3x 50 mg/mL, 2x 5 mg/mL, 1x 1 mg/mL, 1x 0.5 mg/mL, 2x weight-based	Keep SC as it is. (100%)	No Changes.
	Not applicable	12			
	Alternative concentration	22			
	Not completed	11			
Ibuprofen	5 mg/mL	66	3x 4 mg/mL, 2x 2 mg/mL, 2x 6 mg/mL, 1x 0.5 mg/mL, 1x 1 mg/mL, 1x 2.5 mg/mL, 1x 5 mg/mL, 1x weight-based	Keep SC as it is. (100%)	No Changes.
	Not applicable	12			
	Alternative concentration	10			
	Not completed	11			
Indomethacin	0.2 mg/mL	35	10x 0.1 mg/mL, 4x 1 mg/mL, 1x 0.5 mg/mL, 1x 0.05 mg/mL, 3x weight-based	Keep SC as it is. (100%)	No Changes.
	Not applicable	33			
	Alternative concentration	19			
	Not completed	11			
Levetiracetam	10 mg/mL	45	14x 5 mg/mL, 2x 1 mg/mL, 1x 50 mg/mL, 1x 20 mg/mL, 2x weight-based	Keep SC as it is. (67%) Adjusting SC. (33%)	100 mg/mL deleted.
	100 mg/mL	31			
	Not applicable	12			
	Alternative concentration	20			
	Not completed	11			
Meropenem	10 mg/mL	50	14x 50 mg/mL, 10x 5 mg/mL, 8x 20 mg/mL, 1x 4 mg/mL, 1x 25 mg/mL, 1x 100 mg/mL, 1x weight-based	Keep SC as it is. (100%)	No Changes.
	Not applicable	9			
	Alternative concentration	35			
	Not completed	11			
Metamizole Na · 1 H_2O	100 mg/mL	29	10x 10 mg/mL, 3x 20 mg/mL, 3x 50 mg/mL, 3x 500 mg/mL, 1x 5 mg/mL, 1x 200 mg/mL, 3x weight-based	Keep SC as it is. (75%) Not recommending use (25%)	Medication deleted.
	Not applicable	36			
	Alternative concentration	24			
	Not completed	11			
Metronidazole	5 mg/mL	74		Keep SC as it is. (100%)	No Changes.
	Not applicable	12			
	Alternative concentration	0			
	Not completed	11			
Midazolam	0.1 mg/mL	26	8x 0.5 mg/mL, 5x 0.2 mg/mL, 5x 1 mg/mL, 5x 2 mg/mL, 5x 5 mg/mL, 2x 0.1 mg/mL, 1x 0.4 mg/mL, 1x 0.05 mg/mL, 7x weight-based	Keep SC as it is. (92%) Adjusting SC. (8%)	No Changes.
	1 mg/mL	63			
	Not applicable	6			
	Alternative concentration	28			
	Not completed	11			
Milrinone	200 µg/mL	45	9x 100 µg/mL, 5x 120 µg/mL, 4x 50 µg/mL, 3x 200 µg/mL, 2x 30 µg/mL, 2x 40 µg/mL, 2x 60 µg/mL, 2x 400 µg/mL, 1x 80 µg/mL, 1x 150 µg/mL, 1x 300 µg/mL, 1x 800 µg/mL, 1x 1 mg/mL, 13x weight-based	Keep SC as it is. (75%) Adjusting SC. (25%)	50 µg/mL added.
	Not applicable	16			
	Alternative concentration	31			
	Not completed	11			
Morphine salts	10 µg/mL	31	22x 100 µg/mL, 9x 1 mg/mL, 8x 50 µg/mL, 4x 20 µg/mL, 2x 30 µg/mL, 2x 40 µg/mL, 2x 200 µg/mL, 2x 500 µg/mL, 1x 150 µg/mL, 1x 300 µg/mL, 1x 400 µg/mL, 1x 600 µg/mL, 1x 800 µg/mL, 10x weight-based	Keep SC as it is. (50%) Adjusting SC. (50%)	100 µg/mL added.
	200 µg/mL	28			
	Not applicable	10			
	Alternative concentration	49			
	Not completed	11			
Naloxone HCl	100 µg/mL	28	3x 50 µg/mL, 2x 40 µg/mL, 1x 20 µg/mL, 1x 10 µg/mL, 1x weight-based	Keep SC as it is. (100%)	400 µg/mL changed to 40 µg/mL.
	400 µg/mL	43			
	Not applicable	24			
	Alternative concentration	8			
	Not completed	11			
Norepinephrine	10 µg/mL	40	14x 20 µg/mL, 9x 60 µg/mL, 5x 100 µg/mL, 3x 6 µg/mL, 2x 15 µg/mL, 2x 2.5 µg/mL, 1x 4 µg/mL, 1x 5 µg/mL, 1x 12 µg/mL, 1x 25 µg/mL, 7x 30 µg/mL, 1x 200 µg/mL, 1x 1 mg/mL, 13x weight-based	Keep SC as it is. (58%) Adjusting SC. (42%)	20 µg/mL added.
	40 µg/mL	28			
	Not applicable	11			
	Alternative concentration	47			
	Not completed	11			
Paracetamol	10 mg/mL	76	1x 5 mg/mL	Keep SC as it is. (100%)	No Changes.
	Not applicable	9			
	Alternative concentration	1			
	Not completed	11			
Penicillin G	0.1 Mio IU/mL	59	3x 0.2 Mio IE/mL, 1x 1 Mio IE/mL, 1x 0.1 Mio IE/mL, 1x 0.04 Mio IE/mL, 3x weight-based	Keep SC as it is. (100%)	No Changes.
	Not applicable	20			
	Alternative concentration	9			
	Not completed	11			
Phenobarbital (base)	10 mg/mL	60	18x 20 mg/mL, 1x 200 mg/mL, 1x weight-based	Keep SC as it is. (100%)	No Changes.
	Not applicable	9			
	Alternative concentration	20			
	Not completed	11			
Phytomenadione (Vitamin K1)	1 mg/mL	61	7x 10 mg/mL, 3x 0.1 mg/mL, 2x 0.2 mg/mL, 1x 0.5 mg/mL	Keep SC as it is. (83%) Adjusting SC. (17%)	No Changes.
	Not applicable	15			
	Alternative concentration	14			
	Not completed	11			
Piperacillin/ Tazobactam	80/10 mg/mL	47	18x 100/12.5 mg/mL, 5x 200/25 mg/mL, 4x 40/5 mg/mL, 1x 50/6.25 mg/mL, 2x weight-based	Keep SC as it is. (58%) Adjusting SC. (42%)	Changed to 100/12.5 mg/mL.
	Not applicable	12			
	Alternative concentration	30			
	Not completed	11			
Piritramid	0,1 mg/mL	32	1x 0.15 mg/mL, 1x 0.5 mg/mL, 1x 1 mg/mL, 3x weight-based	Keep SC as it is. (92%) Not recommending use (8%)	Medication deleted.
	1 mg/mL	47			
	Not applicable	26			

Medication	Answers	Count	Suggested Concentrations	Expert Evaluation (*n*=12)	Changes
	Alternative concentration	6			
	Not completed	11			
Prednisolone-21-hydrogensuccinate	1 mg/mL	43	9x 5 mg/mL, 5x 10 mg/mL, 3x 50 mg/mL, 3x 2 mg/mL, 2x 25 mg/mL, 2x 12.5 mg/mL, 1x weight-based	Keep SC as it is. (75%) Adjusting SC. (25%)	5 mg/mL added.
	Not applicable	27			
	Alternative concentration	19			
	Not completed	11			
Propofol	10 mg/mL	69	10x 5 mg/mL	Keep SC as it is. (75%) Adjusting SC. (25%)	10 mg/mL changed to 5 mg/mL.
	Not applicable	14			
	Alternative concentration	10			
	Not completed	11			
Sildenafil	0.16 mg/mL	20	2x 0.2 mg/mL, 1x 0.07 mg/mL, 1x 0.8 mg/mL, 1x 1 mg/mL, 1x weight-based	Keep SC as it is. (83%) Adjusting SC. (17%)	No Changes.
	0.8 mg/mL	36			
	Not applicable	45			
	Alternative concentration	6			
	Not completed	11			
Teicoplanin	10 mg/mL	29	4x 20 mg/mL, 3x 4 mg/mL, 2x 5 mg/mL, 2x 40 mg/mL, 2x 66.7 mg/mL, 1x 1 mg/mL, 1x 8 mg/mL, 1x 50 mg/mL, 1x 133.3 mg/mL, 2x weight-based	Keep SC as it is. (100%)	No Changes.
	Not applicable	41			
	Alternative concentration	18			
	Not completed	11			
Theophylline	1 mg/mL	29	9x 4 mg/mL, 6x 10 mg/mL, 4x 2 mg/mL, 2x 1 mg/mL, 2x 40 mg/mL, 1x 0.4 mg/mL, 1x 5 mg/mL, 1x 8 mg/mL, 3x weight-based	Keep SC as it is. (100%)	No Changes.
	Not applicable	37			
	Alternative concentration	24			
	Not completed	11			
Vancomycin HCl	5 mg/mL	68	5x 50 mg/mL, 3x 2.5 mg/mL, 3x 10 mg/mL, 1x 1 mg/mL, 1x 4 mg/mL, 2x weight-based	Keep SC as it is. (100%)	No Changes.
	Not applicable	6			
	Alternative concentration	15			
	Not completed	11			
Vecuronium Br	0.1 mg/mL	26	16x 1 mg/mL, 3x 0.5 mg/mL, 2x 0.2 mg/mL, 2x 0.05 mg/mL, 1x 2 mg/mL, 3x weight-based	Keep SC as it is. (83%) Adjusting SC. (17%)	1 mg/mL added.
	Not applicable	42			
	Alternative concentration	25			
	Not completed	11			

SC: standard concentration, SmPC: summary of product characteristics

Table S3. Information on preparation and administration of the SC Proposal List.

Nr.	Medication	Recommended standard concentration	Reconstitution/ Preparation	Diluent	Physicochemical stability (24 h at RT)	Compatibility with polypropylene syringes	Osmolarity <900 mOsm/L	pH range 5-9
**1**	Aciclovir	5 mg/mL	Dilute 10 mL (250 mg/10 mL [1]) to 50 mL.	NS	✓ [1]	✓ [1]	✓ [2, 3]	X [2, 3]
**2**	Alprostadil	1 µg/mL<br>(1.58 mg/mL ethanol)	Dilute 0.1 mL (500 µg/mL [4]) to 50 mL.	NS/ D5W	✓ [4]	Glas [4]	✓*	✓*
		2 µg/mL<br>(3.16 mg/mL ethanol)	Dilute 0.2 mL (500 µg/mL [3]) to 50 mL.		✓ [4]			
**3**	Amiodarone HCl	1 mg/mL<br>(0.4 mg/mL benzyl alcohol)	Dilute 1 mL (150 mg/3 mL [5]) to 50 mL.	D5W	✓ [2]<br>Protect from light if CI [5]	✓ [2]	✓ [2]	(X) [2]
		5 mg/mL<br>(2 mg/mL benzyl alcohol)	Dilute 5 mL (150 mg/3 mL [5]) to 50 mL.		✓ [2]<br>Protect from light if CI [5]			
**4**	Ampicillin	100 mg/mL	Reconstitute with WFI according to SmPC. [6]	-	For immediate use [2, 6]	?	✓ [2, 3]	! [2, 3]
**5**	Ampicillin/ Sulbactam	100/50 mg/mL	Reconstitute 1,000/500 mg with 3.2 mL WFI. [7]<br>Dilute 4.2 mL (1,000/500 mg) to 10 mL.	NS	For immediate use [2, 7]	?	X*	! [2]
**6**	Cefotaxime	100 mg/mL	Reconstitute with WFI. [8] Dilute 2 mL (250 mg/mL) to 5 mL.	WFI	RT (12 h);<br>✓ at 2-6 °C [9]	(✓) [9]	✓ [2]	✓ [2]
**7**	Ceftazidime	90 mg/mL	Reconstitute 0.5 g with 5 mL NS. [10]	-	RT (6 h);<br>5 °C (12 h) [10]<br>Protect from light [2]	(✓) [9]	✓ [2]	(✓) [2]
**8**	Cefuroxime	116 mg/mL	Reconstitute 250 mg with 2 mL WFI or 750 mg with 6 mL WFI. [11, 12]	-	RT (5h);<br>✓ at 2-8 °C,<br>Protect from light [11]	(✓) [9]	(✓) [2]	✓ [2]
**9**	Clarithromycin	2 mg/mL	Reconstitute according to SmPC. [13]<br>Dilute 2 mL (50 mg/mL) to 50 mL	NS/ D5W	RT (6h);<br>✓ at 2-8 °C,<br>Protect from light [13]	?	✓*	!*
**10**	Clindamycin	5 mg/mL	Dilute 1 mL (150 mg/mL [14]) to 30 mL.	NS/ D5W	✓ [14]	(✓) [9]	✓ [2]	✓ [2]
**11**	Clonidine HCl	1.5 µg/mL	Dilute 0.5 mL (150 µg/mL [15]) to 50 mL.	NS	(✓) [16]	?	✓*	!*
		7.5 µg/mL	Dilute 2.5 mL (150 µg/mL [15]) to 50 mL.		(✓) [16]			
**12**	Caffeine citrate	20 mg/mL	Undiluted. [17]	-	✓ [17]	?	✓ [17]	! [17]
**13**	Dexamethasone-21-dihydrogenphosphate	0.1 mg/mL	Dilute 1 mL (4 mg/mL [18]) to 40 mL.	NS/ D5W	✓ [18]<br>Protect from light [2]	✓ [9]	(✓) [2]	(✓) [18]
		0.5 mg/mL	Dilute 1 mL (4 mg/mL [18]) to 8 mL.		✓ [18]<br>Protect from light [2]			
**14**	Dobutamine	0.5 mg/mL	Dilute 5 mL (5 mg/mL [19]) to 50 mL.	NS/ D5W	✓ [19]	(✓) [9]	(✓) [2]	(X) [2]
		1 mg/mL	Dilute 10 mL (5 mg/mL [19]) to 50 mL.		✓ [19]			
		2.5 mg/mL	Dilute 25 mL (5 mg/mL [19]) to 50 mL.		✓ [19]			
**15**	Dopamine HCl	0.5 mg/mL	Dilute 2.5 mL (50 mg/5 mL [20]) to 50 mL.	NS/ D5W	✓ [20]<br>Protect from light [2]	✓ [9]	(✓) [2]	(X) [2]
		1 mg/mL	Dilute 5 mL (50 mg/5 mL [20]) to 50 mL.		✓ [20]<br>Protect from light [2]			
		2.5 mg/mL	Dilute 12.5 mL (50 mg/5 mL [20]) to 50 mL.		✓ [20]<br>Protect from light [2]			
**16**	Doxapram HCl · 1 H_2O	1 mg/mL	Dilute 2.5 mL (20 mg/mL [21]) to 50 mL.	NS/ D5W	✓ [2]	(✓) [9]	✓*	!*
		2 mg/mL	Dilute 5 mL (20 mg/mL [21]) to 50 mL.		(✓) [2]			
**17**	Epinephrine	10 µg/mL<br>(max 5 µg/mL sodium metabisulfite)	Dilute 0.5 mL (1 mg/mL [22]) to 50 mL.	NS	✓ [9]<br>Protect from light [9]	✓ [9]	✓*	X*
		20 µg/mL	Dilute 1 mL (1 mg/mL [22]) to 50 mL.		✓ [9]<br>Protect from light [9]	✓ [9]		

Nr.	Medication	Recommended standard concentration	Reconstitution/ Preparation	Diluent	Physicochemical stability (24 h at RT)	Compatibility with polypropylene syringes	Osmolarity <900 mOsm/L	pH range 5-9
		(max 10 µg/mL sodium metabisulfite)						
		40 µg/mL<br>(max 20 µg/mL sodium metabisulfite)	Dilute 2 mL (1 mg/mL [22]) to 50 mL.		(✓) [9]<br>Protect from light [9]	(✓) [9]		
**18**	Erythromycin	5 mg/mL	Reconstitute according to SmPC. [23]<br>Dilute 5 mL concentrate (50 mg/mL) to 50 mL.	NS/ D5W	✓ [2]	?	✓ [2]	✓ [23]
**19**	Esketamine	0.5 mg/mL	Dilute 5 mL (5 mg/mL [24]) to 50 mL.	NS/ D5W	✓ [24]	(✓) [9]	(✓) [24]	(!) [24]
		5 mg/mL	Undiluted. [24]		✓ [24]			
**20**	Esmolol HCl	10 mg/mL	Undiluted. [25]	-	✓ [25]	✓(PA/PE) [25]	✓ [25]	! [25]
**21**	Fentanyl	2.5 µg/mL	Dilute 2.5 mL (50 µg/mL [26]) to 50 mL.	NS/ D5W	✓ [26]	(✓) [2] [9]	✓ [2]	(✓) [26]
		5 µg/mL	Dilute 5 mL (50 µg/mL [26]) to 50 mL.		✓ [26]			
		10 µg/mL	Dilute 10 mL (50 µg/mL [26]) to 50 mL.		✓ [26]			
**22**	Flucloxacillin	48.5 mg/mL	Reconstitute with 20 mL WFI according to SmPC. [27]	-	RT (1 h)<br>✓2-8°C [27]<br>Protect from light [9]	(✓) [9]	✓ [2]	✓ [2]
**23**	Fluconazole	2 mg/mL	Undiluted. [28]	-	✓ [28]	?	✓ [28]	! [28]
**24**	Furosemide	0.2 mg/mL	Dilute 1 mL (20 mg/2 mL [29]) to 50 mL.	NS	✓ [30]	(✓) [9]	✓ [3]	✓ [3]
		0.4 mg/mL	Dilute 2 mL (20 mg/2 mL [29]) to 50 mL.		✓ [30]			
		1 mg/mL	Dilute 1 mL (20 mg/2 mL [29]) to 10 mL.		✓ [9]	✓ [9]		
		5 mg/mL	Dilute 2 mL (20 mg/2 mL [29]) to 4 mL.		✓ [9]			
**25**	Gentamicin	2 mg/mL	Dilute 2.5 mL (40 mg/mL [31]) to 50 mL.	NS	(✓) [31, 9]	(✓) [2]	✓ [2]	! [2]
**26**	Heparin Na	1 IU/mL	Dilute 0.5 mL (100 IU/mL) to 50 mL.	NS	✓ [2]	(✓) [9]	✓ [2]	✓ [2]
		100 IU/mL	Dilute 0.2 mL (5000 IU/0.2 mL [32]) to 50 mL.		✓ [2]			
**27**	Human Insulin	0.02 IU/mL	Dilute 1 mL (1 IU/mL) to 50 mL.	NS	(X) RT (6 h) [9]	(✓) [9]	(✓) [2]	(✓) [2]
		0.2 IU/mL	Dilute 10 mL (1 IU/mL) to 50 mL.		(✓) [9]<br>Protect from light [9]			
		1 IU/mL	Dilute 0.5 mL (100 IU/mL [33]) to 50 mL.		✓ [9]			
**28**	Hydrocortisone	1 mg/mL	Reconstitute according to SmPC. [34]<br>Dilute 1 mL (50 mg/mL) to 50 mL.	NS/ D5W	RT (4 h) [34]<br>Protect from light [34]	(✓) [9]	✓*	✓*
**29**	Ibuprofen	5 mg/mL	Undiluted. [35]	-	✓ [36]	?	✓ [2]	✓ [2]
**30**	Indomethacin	0.2 mg/mL	Reconstitute according to SmPC (Import).<br>Dilute 0.4 mL (25 mg/mL) to 50 mL.	NS	(✓) [2, 9]	(✓) [2, 9]	(✓) [37]	✓ [2, 37]
**31**	Levetiracetam	10 mg/mL	Dilute 5 mL (100 mg/mL [38]) to 50 mL.	NS/ D5W	✓ [38]	(✓) [9]	(✓) [2]	✓ [2]
**32**	Meropenem	10 mg/mL	Reconstitute with 10 mL NS or D5W [39]<br>Dilute 10 mL (50 mg/mL) to 50 mL.	NS/ D5W	RT (3 h);<br>✓ 2-8°C [39]	✓ [9]	✓*	✓*
**33**	Metronidazole	5 mg/mL	Undiluted. [40]	-	✓ 2-8°C [40]	?	✓ [41]	! [41]
**34**	Midazolam	0.1 mg/mL	Dilute 1 mL (1 mg/mL [42]) to 10 mL.	NS	(✓) [9]	(✓) [9]	✓ [42]	X [42]
		1 mg/mL	Undiluted. [42]		✓ [9]	✓ [9]		
**35**	Milrinone	50 µg/mL	Dilute 2.5 mL (1 mg/mL [43]) to 50 mL.	NS/ D5W	✓ [43]	(✓) [9]	✓ [2]	(X) [2]
		200 µg/mL	Dilute 10 mL (1 mg/mL [43]) to 50 mL.		✓ [43]	✓ [9]		
**36**	Morphine salts	10 µg/mL	Dilute 5 mL (100 µg/mL) to 50 mL.	NS	(✓) [9]	(✓) [9]	(✓) [2]	(!) [2]
		100 µg/mL	Dilute 0.5 mL (10 mg/mL [44]) to 50 mL.		✓ [44]	✓ [9]		
		200 µg/mL	Dilute 1 mL (10 mg/mL [44]) to 50 mL.		✓ [44]	✓ [9]		
**37**	Naloxone HCl	40 µg/mL	Dilute 1 mL (400 µg/mL [45]) to 10 mL.	NS/ D5W	✓ [45]	?	(✓) [45]	X [45]
		100 µg/mL	Dilute 1 mL (400 µg/mL [45]) to 4 mL.		✓ [45]			
**38**	Norepinephrine	10 µg/mL	Dilute 0.5 mL (1 mg/mL [46]) to 50 mL.	NS/ D5W	✓ 2-8°C [9]<br>Protect from light [9]	✓ [9]	✓ [46]	(!) [2]
		20 µg/mL	Dilute 1 mL (1 mg/mL [46]) to 50 mL.		(✓) [9]	(✓) [9]		

Nr.	Medication	Recommended standard concentration	Reconstitution/ Preparation	Diluent	Physicochemical stability (24 h at RT)	Compatibility with polypropylene syringes	Osmolarity <900 mOsm/L	pH range 5-9
					Protect from light [9]			
		40 µg/mL	Dilute 2 mL (1 mg/mL [46]) to 50 mL.		(✓) [46]<br>Protect from light [9]	✓ [9]		
**39**	Paracetamol	10 mg/mL	Undiluted. [47]	-	✓ [47]	✓ [2]	✓ [47]	✓ [2]
**40**	Penicillin G	0.1 Mio IU/mL	Reconstitute and dilute according to SmPC. [48]	-	X RT (8 h) [48]<br>Protect from light [9]	?	✓ [48]	✓ [48]
**41**	Phenobarbital (base)	10 mg/mL<br>(4 mg/mL ethanol,<br>35 mg/mL propylene glycol)	Dilute 2.5 mL (200 mg/mL [49]) to 50 mL. [2]	NS/ D5W	✓ 2-8°C [2]	(✓) [9]	(✓) [2]	(✓) [2]
**42**	Phytomenadione<br>(Vitamin K1)	1 mg/mL	Dilute 1 mL (10 mg/mL [50]) to 10 mL. [2]	NS/ D5W	For immediate use [2]<br>Protect from light [2]	?	✓ [2]	(✓) [2]
**43**	Piperacillin/Tazobactam	100/12.5 mg/mL	Reconstitute according to SmPC. [51]<br>Dilute 10 mL (200/25 mg/mL) to 20 mL.	NS/ D5W	RT (12 h)<br>(✓) 2-8°C [51]	(✓) [9]	(✓) [2]	(✓) [2]
**44**	Prednisolone-21-hydrogensuccinate	1 mg/mL	Dilute 1 mL (5 mg/mL [52]) to 5 mL.	NS/ D5W	RT (6 h) [52]	?	(✓) [2]	(✓) [2]
		5 mg/mL	Reconstitute according to SmPC. [52]		RT (6 h) [52]			
**45**	Propofol	5 mg/mL	Undiluted. [53]	-	(✓) [9]	(✓) [9]	(✓) [2]	(✓) [2]
**46**	Sildenafil	0.16 mg/mL	Dilute 10 mL (0.8 mg/mL [54]) to 50 mL.	NS/ D5W	(✓) [9]	(✓) [9]	✓*	X*
		0.8 mg/mL	Undiluted. [54]		✓ [9]			
**47**	Teicoplanin	10 mg/mL	Reconstitute according to SmPC. [55]<br>Dilute 3 mL (200 mg) to 20 mL.	NS/ D5W	✓ 2-8 °C [55]	?	(✓) [2]	(✓) [2]
**48**	Theophylline	1 mg/mL	Dilute 1 mL (40 mg/mL [56]) to 40 mL.	NS/ D5W	✓ [56]	?	✓*	✓*
**49**	Vancomycin HCl	5 mg/mL	Reconstitute according to SmPC. [57]<br>Dilute 5 mL (50 mg/mL) to 50 mL.	NS/ D5W	✓ [9]	✓ [9]	✓ [2, 3]	X [3]
**50**	Vecuronium Br	0.1 mg/mL	Reconstitute with 5 mL WFI. [58]<br>Dilute 2.5 mL (2 mg/mL) to 50 mL.	NS/ D5W	✓ [58]<br>Protect from light [2]	(✓) [2] [9]	(✓) [58]	(!) [2]
		1 mg/mL	Reconstitute with 5 mL WFI. **[58]**<br>Dilute 5 mL (2 mg/mL) to 10 mL.		✓ [58]<br>Protect from light [2]			

✓: specification fulfilled, !: specification at the limit, X: specification not fulfilled, ?: no data available, ():estimated information, *: own measurement result, D5W: dextrose in water 5%, NS: 0.9% sodium chloride infusion solution, RT: room temperature (20-25 °C), SmPC: summary of product characteristics, WFI: sterile water for injection.

References – Table S3

[1] Accord Healthcare B.V., *SmPC: Aciclovir Accord 25 mg/ml Konzentrat zur Herstellung einer Infusionslösung,* July 2024.

[2] Bundesverband Deutscher Krankenhausapotheker e.V. (ADKA), "ADKA Arzneimittel-Info-Datenbank," [Online]. Available: https://www.adka-aminfo.de/. [Accessed 11 December 2025].

[3] L. Torralba-Fernández, M. García-Palomo, M. López de Abechuco-Ruiz et al., "Standarization and characterization of intravenous drug dilutions in critically ill pediatric patients," *Farmacia Hospitalaria,* vol. 49, no. 6, pp. T373-T379, 2025.

[4] Pfizer PHARMA GmbH, *SmPC: MINPROG 500 μg,* November 2023.

[5] Sanofi-Aventis Deutschland GmbH, *SmPC: Cordarex Injektionslösung,* August 2022.

[6] Ratiopharm GmbH, *SmPC: Ampicillin-ratiopharm 0,5 g/1,0 g/2,0 g/5,0 g,* September 2024.

[7] Fresenius Kabi Deutschland GmbH, *SmPC: Ampicillin/Sulbactam Kabi,* October 2025.

[8] Fresenius Kabi Deutschland GmbH, *SmPC: Cefotaxim Fresenius 0,5 g Pulver zur Herstellung einer Injektionslösung,* April 2024.

[9] "Stabilis.org," [Online]. Available: www.stabilis.org. [Accessed 12 December 2025].

[10] Fresenius Kabi Deutschland GmbH, *SmPC: Ceftazidim Kabi 0,5 g/1 g,* September 2024.

[11] Fresenius Kabi Deutschland GmbH, *SmPC: Cefuroxim Fresenius 250 mg Pulver zur Herstellung einer Injektionslösung,* October 2023.

[12] Fresenius Kabi Deutschland GmbH, *SmPC: Cefuroxim Fresenius 750 mg Pulver zur Herstellung einer Injektionslösung,* October 2023.

[13] Hameln Pharma Gmbh, *SmPC: Clarithromycin-hameln 500 mg Pulver für ein Konzentrat zur Herstellung einer Infusionslösung,* February 2025.

[14] Ratiopharm GmbH, *SmPC: Clindamycin-ratiopharm Injektionslösung,* April 2024.

[15] Glenwood GmbH, *SmPC: Catapresan,* December 2021.

[16] ratiopharm GmbH, *SmPC: Clonidin-ratiopharm Ampullen,* October 2024.

[17] Chiesi GmbH, *SmPC: Peyona 20 mg/ml Infusionslösung und Lösung zum Einnehmen,* March 2024.

[18] Merck Healthcare Germany GmbH, *SmPC: Fortecortin Inject 4 / 8 / 40 / 100 mg,* Februar 2022.

[19] Hameln Pharma GmbH, *SmPC: Dobutamin-hameln 5 mg/ml,* June 2024.

[20] Fresenius Kabi Deutschland GmbH, *SmPC: Dopamin Fresenius 50 mg/5 ml,* July 2018.

[21] Carinopharm GmbH, *SmPC: Dopram,* September 2021.

[22] Cheplapharm Arzneimittel GmbH, *SmPC: Suprarenin Ampullen,* June 2021.

[23] Panpharma GmbH, *SmPC: Erythromycin Panpharma 500 mg Pulver zur Herstellung einer Infusionslösung,* March 2024.

[24] Inresa Arzneimittel GmbH, *SmPC: Esketamin Inresa 5/25 mg/ml Ampullen,* September 2022.

[25] Baxter Deutschland GmbH, *SmPC: Brevibloc 10 mg/ml Infusionslösung,* May 2018.

[26] Hameln Pharma GmbH, *SmPC: Fentanyl-hameln 50 Mikrogramm/ml Injektionslösung,* March 2024.

[27] Ibigen Srl, *SmPC: Flucloxacillin Ibisqus 1.000 mg Pulver zur Herstellung einer Injektions-/Infusionslösung,* December 2017.

[28] Fresenius Kabi Deutschland GmbH, *SmPC: Fluconazol Kabi 2 mg/ml Infusionslösung,* March 2024.

[29] Sanofi-Aventis Deutschland GmbH, *SmPC: Lasix® 20 mg Injektionslösung,* September 2024.

[30] American Society of Health-System Pharmacists, ASHP® Injectable Drug Information: A Comprehensive Guide to Compatibility and Stability, https://doi.org/10.37573/9781585287444, 2025.

[31] Ratiopharm GmbH, *SmPC: Gentamicin-ratiopharm SF,* June 2024.

[32] Ratiopharm GmbH, *SmPC: Heparin-Natrium-5000-ratiopharm (Ampullen/Fertigspritzen),* March 2020.

[33] Lilly Deutschland GmbH, *SmPC: Huminsulin,* November 2023.

[34] Pfizer-Pharma GmbH, *SmPC: HYDROCORTISON Pfizer 100 mg/ 250 mg sine,* November 2023.

[35] Recordati Rare Diseases, *SmPC: Pedea 5 mg/ml Injektionslösung,* May 2025.

[36] S. E. Walker, J. Choudhury, S. Law et al., "Stability of Ibuprofen Solutions in Normal Saline or 5% Dextrose in Water," *Can J Hosp Pharm,* vol. 64, no. 5, pp. 354-361, 2011.

[37] Nobelpharma Co., Ltd., *SmPC Indacin IV 1 mg,* September 2023.

[38] UCB Pharma GmbH, *SmPC: Keppra 100 mg/ml Konzentrat zur Herstellung einer Infusionslösung,* December 2024.

[39] Inresa Arzneimittel GmbH, *SmPC: Meropenem Inresa 500_1000 mg,* October 2020.

[40] B. Braun Melsungen AG, *SmPC: Metronidazol B. Braun 5 mg/ml Infusionslösung,* November 2022.

[41] Fresenius Kabi Deutschland GmbH, *SmPC: Metronidazol Fresenius 500 mg/100 ml Infusionslösung,* October 2025.

[42] Hameln Pharma GmbH, *SmPC: Midazolam-hameln,* August 2023.

[43] Stragen Pharma GmbH, *SmPC: Milrinon Stragen 1 mg/ml Konzentrat zur Herstellung einer Infusionslösung,* November 2024.

[44] Hameln Pharma GmbH, *SmPC: Morphin-hameln 10/15/20/100/200 mg Injektionslösung,* November 2023.

[45] B. Braun Melsungen AG, *SmPC: Naloxon B. Braun 0,4 mg/ml Injektions-/Infusionslösung,* December 2017.

[46] Fresenius Kabi Deutschland GmbH, *SmPC: Noradrenalin Kabi 1 mg/ml Konzentrat zur Herstellung einer Infusionslösung,* October 2021.

[47] B. Braun Melsungen AG, *SmPC: Paracetamol B. Braun 10 mg/ml Infusionslösung,* July 2022.

[48] Infectopharm Arzneimittel und Consilium GmbH, *SmPC: Penicillin G INFECTOPHARM 1/5/10 Mega,* March 2025.

[49] Desitin Arzneimittel GmbH, *SmPC: Luminal Injektionslösung,* April 2023.

[50] Cheplapharm Arzneimittel GmbH, *SmPC: Konakion MM 10 mg,* June 2018.

[51] Fresenius Kabi Deutschland GmbH, *SmPC: Piperacillin/Tazobactam Kabi 2 g/0,25 g Pulver zur Herstellung einer Infusionslösung,* November 2021.

[52] Mibe GmbH Arzneimittel, *SmPC: Prednisolut,* November 2023.

[53] B. Braun Melsungen AG, *SmPC: Propofol-®Lipuro 5 mg/ml,* July 2024.

[54] Viatris Healthcare GmbH, *SmPC: Revatio 0,8 mg/ml Injektionslösung,* August 2024.

[55] Ibigen S.r.l., *SmPC: Teicoplanin Ibisqus 200 mg Pulver zur Herstellung einer Injektions-/Infusionslösung oder einer Lösung zum Einnehmen,* February 2020.

[56] Glenwood GmbH Pharmazeutische Erzeugnisse, *SmPC: afpred forte-THEO 200 mg Injektionslösung,* April 2019.

[57] MIP Pharma GmbH, *SmPC: Vanco-saar 500 mg / 1 g,* December 2020.

[58] Inresa Arzneimittel GmbH, *SmPC: Vecuronium Inresa 10 mg,* August 2014.

Table S4. Information on Dosing, Infusion Rates and Infusion Volumes of the SC proposal list.

Nr.	Medication	Dosing ranges per body weight (based on Kinderformularium.DE)	Type of administration	Recommended standard concentration	Weight-based inf. rate per hour or single dose	Weight-based infusion volume per day
1	Aciclovir	30-60 mg/kg/d in 3 doses	short infusion (1 h)	5 mg/mL	2-4 mL/kg/h	6-12 mL/kg/d
2	Alprostadil	5-100 ng/kg/min	continuous infusion	1 µg/mL	0.3-6 mL/kg/h	7.2-144 mL/kg/d
				2 µg/mL	0.15-3 mL/kg/h	3.6-72 mL/kg/d
3	Amiodarone HCl	5-15µg/kg/min	continuous infusion	1 mg/mL	0.3-0.9 mL/kg/h	7.2-21.6 mL/kg/d
		5 mg/kg/dose	bolus	5 mg/mL	1 mL/kg/dose	1 mL/kg/dose
4	Ampicillin	60-400 mg/kg/d in 2-4 doses	bolus	100 mg/mL	0.3-1 mL/kg/dose	0.6-4 mL/kg/d
5	Ampicillin/ Sulbactam	50/25-100/50 mg/kg/d in 2-4 doses	bolus	100/50 mg/mL	0.25 mL/kg/dose	0.5-1 mL/kg/d
6	Cefotaxime	100-200 mg/kg/d in 2-4 doses	bolus	100 mg/mL	0.5 mL/kg/dose	1-2 mL/kg/d
			short infusion (1 h)	100 mg/mL	0.5 mL/kg/h	1-2 mL/kg/d
7	Ceftazidime	50-200 mg/kg/d in 2-6 doses	bolus	90 mg/mL	0.28-0.56 mL/kg/dose	0.56-2.22 mL/kg/d
			short infusion (1 h)	90 mg/mL	0.28-0.56 mL/kg/h	0.56-2.22 mL/kg/d
8	Cefuroxime	50-200 mg/kg/d in 2-6 doses	bolus	116 mg/mL	0.22-0.43 mL/kg/dose	0.44-1.72 mL/kg/d
9	Clarithromycin	15-30 mg/kg/d in 2 doses	short infusion (1 h)	2 mg/mL	3.75-7.5 mL/kg/h	7.5-15 mL/kg/d
10	Clindamycin	10-40 mg/kg/d in 2-4 doses	short infusion (1 h)	5 mg/mL	1-4 mL/kg/h	2-8 mL/kg/d
11	Clonidine HCl	0.5-3 µg/kg/h	continuous infusion	1.5 µg/mL	0.33-2 mL/kg/h	8-48 mL/kg/d
				7.5 µg/mL	0.07-0.4 mL/kg/h	1.6-9.6 mL/kg/d
12	Caffeine citrate	5-20 mg/kg/d in 1-2 doses	bolus (10 min)	20 mg/mL	0.25-0.5 mL/kg/dose	0.25-1 mL/kg/d
			short infusion (30 min)	20 mg/mL	1-2 mL/kg/h	0.25-1 mL/kg/d
13	Dexamethasone-21-dihydrogenphosphate	0.3-0.5 mg/kg/d	bolus	0.1 mg/mL	0.75-5 mL/kg/dose	3-5 mL/kg/d
				0.5 mg/mL	0.15-1 mL/kg/dose	0.6-1 mL/kg/d
14	Dobutamine	2-20 µg/kg/min	continuous infusion	0.5 mg/mL	0.24-2.4 mL/kg/h	5.76-57.6 mL/kg/d
				1 mg/mL	0.12-1.2 mL/kg/h	2.88-28.8 mL/kg/d
				2.5 mg/mL	0.048-0.48 mL/kg/h	1.15-11.5 mL/kg/d
15	Dopamine HCl	2-20 µg/kg/min	continuous infusion	0.5 mg/mL	0.24-2.4 mL/kg/h	5.76-57.6 mL/kg/d
				1 mg/mL	0.12-1.2 mL/kg/h	2.88-28.8 mL/kg/d
				2.5 mg/mL	0.048-0.48 mL/kg/h	1.15-11.5 mL/kg/d
16	Doxapram HCl · 1 H_2O	0.5-2 mg/kg/h	continuous infusion	1 mg/mL	0.5-2 mL/kg/h	12-48 mL/kg/d
				2 mg/mL	0.25-1 mL/kg/h	6-24 mL/kg/d
17	Epinephrine	0.05*-0.5 µg/kg/min	continuous infusion	10 µg/mL	0.12-3 mL/kg/h	2.88-72 mL/kg/d
				20 µg/mL	0.06-1.5 mL/kg/h	1.44-36 mL/kg/d
				40 µg/mL	0.03-0.75 mL/kg/h	0.72-18 mL/kg/d
18	Erythromycin	20-50 mg/kg/d in 2-4 doses	short infusion (1 h)	5 mg/mL	2-3 mL/kg/h	4-10 mL/kg/d
19	Esketamine	0.25-1 mg/kg/dose	bolus	0.5 mg/mL	0.5-2 mL/kg/dose	0.5-2 mL/kg/dose
				5 mg/mL	0.05-0.2 mL/kg/dose	0.05-0.2 mL/kg/dose
		0.5-3 mg/kg/h	continuous infusion	0.5 mg/mL	1-6 mL/kg/h	24-144 mL/kg/d
				5 mg/mL	0.1-0.6 mL/kg/h	2.4-14.4 mL/kg/d
20	Esmolol HCl	50*-200* µg/kg/min	continuous infusion	10 mg/mL	0.3-1.2 mL/kg/h	7.2-28.8 mL/kg/d
21	Fentanyl	0.5-10 µg/kg/h	continuous infusion	2.5 µg/mL	0.2-4 mL/kg/h	4.8-96 mL/kg/d
				5 µg/mL	0.1-2 mL/kg/h	2.4-48 mL/kg/d
				10 µg/mL	0.05-1 mL/kg/h	1.2-24 mL/kg/d
22	Flucloxacillin	50-200 mg/kg/d in 2-6 doses	short infusion (1 h)	48.5 mg/mL	0.52 mL/kg/h	1.03-4.12 mL/kg/d
23	Fluconazole	3-25 mg/kg/d in 1 dose	short infusion (1 h)	2 mg/mL	1.5-12.5 mL/kg/h	1.5-12.5 mL/kg/d
24	Furosemide	4*-8* mg/kg/d	bolus	1 mg/mL	0.5-2 mL/kg/dose	0.5-2 mL/kg/dose
				5 mg/mL	0.1-0.4 mL/kg/dose	0.1-0.4 mL/kg/dose
			continuous infusion	0.2 mg/mL	0.83-1.67 mL/kg/h	20-40 mL/kg/d
				0.4 mg/mL	0.42-0.83 mL/kg/h	10-20 mL/kg/d
				1 mg/mL	0.17-0.33 mL/kg/h	4-8 mL/kg/d
25	Gentamicin	4-7.5 mg/kg/d in 1 dose	short infusion (30 min)	2 mg/mL	4-7.5 mL/kg/h	2-3.75 mL/kg/d
26	Heparin Na	5-10 IU/kg/h	continuous infusion	1 IU/mL	5-10 mL/kg/h	120-240 mL/kg/d

Nr.	Medication	Dosing ranges per body weight (based on Kinderformularium.DE)	Type of administration	Recommended standard concentration	Weight-based inf. rate per hour or single dose	Weight-based infusion volume per day
				100 IU/mL	0.05-0.1 mL/kg/h	1.2-2.4 mL/kg/d
**27**	Human Insulin	0.013-0.2 IU/kg/h	continuous infusion	0.02 IU/mL	0.65-10 mL/kg/h	15.6-240 mL/kg/d
				0.2 IU/mL	0.065-1 mL/kg/h	1.56-24 mL/kg/d
				1 IU/mL	0.013-0.2 mL/kg/h	0.31-4.8 mL/kg/d
**28**	Hydrocortisone	0.5-5 mg/kg/d in 1-4 doses	bolus	1 mg/mL	0.5-5 mL/kg/dose	0.5-5 mL/kg/d
**29**	Ibuprofen	5-40 mg/kg/d in 1-4 doses	short infusion (1 h)	5 mg/mL	1-3.6 mL/kg/h	1-8 mL/kg/d
**30**	Indomethacin	0.2-0.5 mg/kg/d in 1-2 doses	short infusion (1 h)	0.2 mg/mL	1-2.5 mL/kg/h	1-2.5 mL/kg/d
**31**	Levetiracetam	10-60 mg/kg/d in 2 doses	short infusion (30 min)	10 mg/mL	1-8 mL/kg/h	1-6 mL/kg/d
**32**	Meropenem	40-120 mg/kg/d in 2-3 doses	short infusion (30 min)	10 mg/mL	4-8 mL/kg/h	4-12 mL/kg/d
			short infusion (2 h)	10 mg/mL	1-2 mL/kg/h	4-12 mL/kg/d
**33**	Metronidazole	7.5-30 mg/kg/d in 1-2 doses	short infusion (30 min)	5 mg/mL	3-12 mL/kg/h	1.5-6 mL/kg/d
**34**	Midazolam	0.05-0.2 mg/kg/dose	bolus	0.1 mg/mL	0.5-2 mL/kg/dose	0.5-2 mL/kg/dose
				1 mg/mL	0.05-0.2 mL/kg/dose	0.05-0.2 mL/kg/dose
		0.05-0.4 mg/kg/h	continuous infusion	0.1 mg/mL	0.5-4 mL/kg/h	12-96 mL/kg/d
				1 mg/mL	0.05-0.4 mL/kg/h	1.2-9.6 mL/kg/d
**35**	Milrinone	0.15-1.25 µg/kg/min	continuous infusion	50 µg/mL	0.18-1.5 mL/kg/h	4.32-36 mL/kg/d
				200 µg/mL	0.045-0.375 mL/kg/h	1.08-9 mL/kg/d
**36**	Morphine salts	10-100 µg/kg/dose	bolus	100 µg/mL	0.1-1 mL/kg/dose	0.1-1 mL/kg/dose
				200 µg/mL	0.05-0.5 mL/kg/dose	0.05-0.5 mL/kg/dose
		3-40 µg/kg/h	continuous infusion	10 µg/mL	0.3-4 mL/kg/h	7.2-96 mL/kg/d
				100 µg/mL	0.03-0.4 mL/kg/h	0.72-9.6 mL/kg/d
				200 µg/mL	0.015-0.2 mL/kg/h	0.36-4.8 mL/kg/d
**37**	Naloxone HCl	10-100* µg/kg/dose	bolus	40 µg/mL	0.25-2.5 mL/kg/dose	0.25-2.5 mL/kg/dose
				100 µg/mL	0.1-1 mL/kg/dose	0.1-1 mL/kg/dose
**38**	Norepinephrine	0.05-2 µg/kg/min	continuous infusion	10 µg/mL	0.3-12 mL/kg/h	7.2-288 mL/kg/d
				20 µg/mL	0.15-6 mL/kg/h	3.6-144 mL/kg/d
				40 µg/mL	0.075-3 mL/kg/h	1.8-72 mL/kg/d
**39**	Paracetamol	12-60 mg/kg/d in 1-4 doses	short infusion (30 min)	10 mg/mL	1.2-4 mL/kg/h	1.2-6 mL/kg/d
**40**	Penicillin G	50,000-400,000 IU/kg/d in 2-4 doses	bolus	0.1 Mio IU/mL	0.25-0.5 mL/kg/dose	0.5-4 mL/kg/d
**41**	Phenobarbital (base)	2.5-20 mg/kg/d in 1-2 doses	bolus	10 mg/mL	0.25-2 mL/kg/dose	0.25-2 mL/kg/d
			short infusion (30 min)	10 mg/mL	0.5-4 mL/kg/h	0.25-2 mL/kg/d
**42**	Phytomenadione (Vitamin K1)	0.2 mg/kg/dose	bolus	1 mg/mL	0.2 mL/kg/dose	0.2 mL/kg/dose
**43**	Piperacillin/Tazobactam	320/40-400/50 mg/kg/d in 4 doses	short infusion (30 min)	100/12.5 mg/mL	1.6-2 mL/kg/h	3.2-4 mL/kg/d
			short infusion (3 h)	100/12.5 mg/mL	0.27-0.33 mL/kg/h	3.2-4 mL/kg/d
**44**	Prednisolone-21-hydrogensuccinate	0.25-2 mg/kg/d in 1-3 doses	bolus	1 mg/mL	0.25-2 mL/kg/dose	0.5-2 mL/kg/d
				5 mg/mL	0.05-0.4 mL/kg/dose	0.1-0.4 mL/kg/d
**45**	Propofol	0.5-4 mg/kg/dose	bolus	5 mg/mL	0.1-0.8 mL/kg/dose	0.1-0.8 mL/kg/dose
**46**	Sildenafil	0.4 mg/kg/dose	bolus	0.8 mg/mL	0.5 mL/kg/dose	0.5 mL/kg/dose
			short infusion (3 h)	0.8 mg/mL	0.17 mL/kg/h	0.5 mL/kg/dose
		1.6 mg/kg/d	continuous infusion	0.16 mg/mL	0.42 mL/kg/h	10 mL/kg/d
**47**	Teicoplanin	6-16 mg/kg/d in 1 dose	short infusion (30 min)	10 mg/mL	1.2-3.2 mL/kg/h	0.6-1.6 mL/kg/d
**48**	Theophylline	Initial: 5-10 mg/kg/dose<br>0.4-0.7 mg/kg/h	continuous infusion	1 mg/mL	0.4-0.7 mL/kg/h<br>Initial: 5-10 mL/kg/dose	9.6-16.8 mL/kg/d
**49**	Vancomycin HCl	20-60 mg/kg/d in 2-4 doses	short infusion (1 h)	5 mg/mL	1.6-3 mL/kg/h	4-12 mL/kg/d
			short infusion (2 h)	5 mg/mL	0.8-1.5 mL/kg/h	4-12 mL/kg/d
**50**	Vecuronium Br	0.01-0.1 mg/kg/dose	bolus	0.1 mg/mL	0.1-1 mL/kg/dose	0.1-1 mL/kg/dose
				1 mg/mL	0.01-0.1 mL/kg/dose	0.01-0.1 mL/kg/dose
			continuous infusion	0.1 mg/mL	0.5-1 mL/kg/h	12-24 mL/kg/d
				1 mg/mL	0.05-0.1 mL/kg/h	1.2-2.4 mL/kg/d

Kg: body weight in kilogram. * dosing range was adjusted to practical evidence and does not correlate with the dosing proposal of Kinderformularium.DE.

Figure S1. Nationwide survey on the SC proposal list (printed version; survey translated from German).

UNIKLINIK RWTH AACHEN | ADKA BUNDESVERBAND DEUTSCHER KRANKENHAUSAPOTHEKER E.V. | GNPI

Standard Concentrations for Intravenous Medications in Neonatology

Please take a moment to review, together with nurses, physicians, and pharmacists, the listed standard concentrations (SC), and evaluate their potential use in your clinic. In some cases, multiple concentrations per medication and routes of administration are proposed to accommodate the weight ranges and needs of preterm and newborn infants (500 g to 5 kg).

The paper version is intended as a worksheet. Please transfer the final evaluation to the online survey to submit the data.

Medication	Recommended Standard Concentration (Preparation*)	Routes of Administration	Could this standard concentration be used in your clinic?
**Aciclovir**	5 mg/mL	short infusion	☐ Yes<br>☐ Not applicable<br>☐ Alternative SC with reason:
**Alprostadil**	1 µg/mL<br>2 µg/mL	continuous infusion<br>continuous infusion	☐ Yes<br>☐ Yes<br>☐ Not applicable<br>☐ Alternative SC with reason:
**Amiodarone HCl**	5 mg/mL	bolus	☐ Yes<br>☐ Not applicable<br>☐ Alternative SC with reason:
**Ampicillin**	100 mg/mL	bolus	☐ Yes<br>☐ Not applicable<br>☐ Alternative SC with reason:
**Ampicillin/ Sulbactam**	250/ 125 mg/mL	bolus	☐ Yes<br>☐ Not applicable<br>☐ Alternative SC with reason:
**Cefotaxime**	50 mg/mL	bolus/ short infusion	☐ Yes<br>☐ Not applicable<br>☐ Alternative SC with reason:
**Ceftazidime**	30 mg/mL<br>90 mg/mL<br>(500 mg + 5 mL WFI)*	short infusion<br>bolus	☐ Yes<br>☐ Yes<br>☐ Not applicable<br>☐ Alternative SC with reason:
**Cefuroxime**	116 mg/mL<br>(250 mg + 2 mL WFI)*	bolus	☐ Yes<br>☐ Not applicable<br>☐ Alternative SC with reason:
**Clarithromycin**	2 mg/mL	short infusion	☐ Yes

* For ceftazidime and cefuroxime, volume expansion occurs during reconstitution. The resulting concentration is slightly lower than the expected concentration. The concentrations listed here always refer to the actual concentration obtained during preparation.
** Manufacturers has been asked whether stability data for dilutions are available.
*** The hydrochloride and sulfate salts differ only minimally in the amount of morphine they contain.

Abbreviations

SC: standard concentration, WFI: sterile water for injection, Br: bromide, HCl: hydrochloride, Na: sodium, x1H_2O: hydrate

			☐ Not applicable<br>☐ Alternative SC with reason:
**Clindamycin**	5 mg/mL	short infusion	☐ Yes<br>☐ Not applicable<br>☐ Alternative SC with reason:
**Clonidine HCl**	1.5 µg/mL<br>7.5 µg/mL	continuous infusion<br>continuous infusion	☐ Yes<br>☐ Yes<br>☐ Not applicable<br>☐ Alternative SC with reason:
**Caffeine citrate**	20 mg/mL	bolus/ short infusion	☐ Yes<br>☐ Not applicable<br>☐ Alternative SC with reason:
**Dexamethasone-21-dihydrogen-phosphate**	0.1 mg/mL<br>0.5 mg/mL	bolus<br>bolus	☐ Yes<br>☐ Yes<br>☐ Not applicable<br>☐ Alternative SC with reason:
**Dobutamine**	1 mg/mL	continuous infusion	☐ Yes<br>☐ Not applicable<br>☐ Alternative SC with reason:
**Dopamine HCl**	1 mg/mL	continuous infusion	☐ Yes<br>☐ Not applicable<br>☐ Alternative SC with reason:
**Doxapram HCl x $1H_2O$**	2.5 mg/mL	continuous infusion	☐ Yes<br>☐ Not applicable<br>☐ Alternative SC with reason:
**Epinephrine**	10 µg/mL<br>40 µg/mL	continuous infusion<br>continuous infusion	☐ Yes<br>☐ Yes<br>☐ Not applicable<br>☐ Alternative SC with reason:
**Erythromycin**	5 mg/mL	short infusion	☐ Yes<br>☐ Not applicable<br>☐ Alternative SC with reason:
**Esketamine**	0.5 mg/mL<br>5 mg/mL	bolus/ continuous infusion<br>bolus/ continuous infusion	☐ Yes<br>☐ Yes<br>☐ Not applicable<br>☐ Alternative SC with reason:
**Esmolol HCl**	10 mg/mL	continuous infusion	☐ Yes<br>☐ Not applicable<br>☐ Alternative SC with reason:

* For ceftazidime and cefuroxime, volume expansion occurs during reconstitution. The resulting concentration is slightly lower than the expected concentration. The concentrations listed here always refer to the actual concentration obtained during preparation.
** Manufacturers has been asked whether stability data for dilutions are available.
*** The hydrochloride and sulfate salts differ only minimally in the amount of morphine they contain.
<u>Abbreviations</u>
SC: standard concentration, WFI: sterile water for injection, Br: bromide, HCl: hydrochloride, Na: sodium, $x1H_2O$: hydrate

**Fentanyl**	2.5 µg/mL<br>5 µg/mL<br>10 µg/ mL	bolus/ continuous infusion<br>bolus/ continuous infusion<br>bolus/ continuous infusion	☐ Yes<br>☐ Yes<br>☐ Yes<br>☐ Not applicable<br>☐ Alternative SC with reason:
**Flucloxacillin**	50 mg/mL	short infusion	☐ Yes<br>☐ Not applicable<br>☐ Alternative SC with reason:
**Fluconazole**	2 mg/mL	short infusion	☐ Yes<br>☐ Not applicable<br>☐ Alternative SC with reason:
**Furosemide**	1 mg/mL<br>5 mg/mL<br>0.2 mg/mL<br>0.4 mg/mL	bolus<br>bolus<br>continuous infusion<br>continuous infusion	☐ Yes<br>☐ Yes<br>☐ Yes<br>☐ Yes<br>☐ Not applicable<br>☐ Alternative SC with reason:
**Gentamicin**	2 mg/mL	short infusion	☐ Yes<br>☐ Not applicable<br>☐ Alternative SC with reason:
**Heparin Na**	1 IU/mL<br>100 IU/mL	continuous infusion<br>continuous infusion	☐ Yes<br>☐ Yes<br>☐ Not applicable<br>☐ Alternative SC with reason:
**Human Insulin**	0.002 IU/mL<br>0.2 IU/mL<br>1 IU/mL	continuous infusion<br>continuous infusion<br>continuous infusion	☐ Yes<br>☐ Yes<br>☐ Yes<br>☐ Not applicable<br>☐ Alternative SC with reason:
**Hydrocortisone**	1 mg/mL	bolus	☐ Yes<br>☐ Not applicable<br>☐ Alternative SC with reason:
**Ibuprofen**	5 mg/mL	short infusion	☐ Yes<br>☐ Not applicable<br>☐ Alternative SC with reason:
**Indomethacin**	0.2 mg/mL	short infusion	☐ Yes<br>☐ Not applicable<br>☐ Alternative SC with reason:
**Phytomenadione (Vitamin K_1)**	1 mg/mL **	bolus	☐ Yes<br>☐ Not applicable

* For ceftazidime and cefuroxime, volume expansion occurs during reconstitution. The resulting concentration is slightly lower than the expected concentration. The concentrations listed here always refer to the actual concentration obtained during preparation.
** Manufacturers has been asked whether stability data for dilutions are available.
*** The hydrochloride and sulfate salts differ only minimally in the amount of morphine they contain.

Abbreviations

SC: standard concentration, WFI: sterile water for injection, Br: bromide, HCl: hydrochloride, Na: sodium, $x1H_2O$: hydrate

			☐ Alternative SC with reason:
**Levetiracetam**	10 mg/mL<br>100 mg/mL	short infusion<br>short infusion	☐ Yes<br>☐ Yes<br>☐ Not applicable<br>☐ Alternative SC with reason:
**Meropenem**	10 mg/mL	short infusion, also prolonged	☐ Yes<br>☐ Not applicable<br>☐ Alternative SC with reason:
**Metamizole Na x $1H_2O$**	100 mg/mL	bolus/ short infusion	☐ Yes<br>☐ Not applicable<br>☐ Alternative SC with reason:
**Metronidazole**	5 mg/mL	short infusion	☐ Yes<br>☐ Not applicable<br>☐ Alternative SC with reason:
**Midazolam**	0.1 mg/mL<br>1 mg/mL	bolus<br>bolus	☐ Yes<br>☐ Yes<br>☐ Not applicable<br>☐ Alternative SC with reason:
**Milrinone**	200 µg/mL	continuous infusion	☐ Yes<br>☐ Not applicable<br>☐ Alternative SC with reason:
**Morphine sulfate/ HCl*** **	10 µg/mL<br>200 µg/mL	continuous infusion<br>bolus/ continuous infusion	☐ Yes<br>☐ Yes<br>☐ Not applicable<br>☐ Alternative SC with reason:
**Naloxone HCl**	100 µg/mL<br>400 µg/mL	bolus<br>bolus	☐ Yes<br>☐ Yes<br>☐ Not applicable<br>☐ Alternative SC with reason:
**Norepinephrine**	10 µg/mL<br>40 µg/mL	continuous infusion<br>continuous infusion	☐ Yes<br>☐ Yes<br>☐ Not applicable<br>☐ Alternative SC with reason:
**Paracetamol**	10 mg/mL	short infusion	☐ Yes<br>☐ Not applicable<br>☐ Alternative SC with reason:
**Penicillin G**	0.1 Mio IU/mL	bolus	☐ Yes<br>☐ Not applicable<br>☐ Alternative SC with reason:
**Phenobarbital (base)**	10 mg/mL	bolus/ short infusion	☐ Yes

* For ceftazidime and cefuroxime, volume expansion occurs during reconstitution. The resulting concentration is slightly lower than the expected concentration. The concentrations listed here always refer to the actual concentration obtained during preparation.
** Manufacturers has been asked whether stability data for dilutions are available.
*** The hydrochloride and sulfate salts differ only minimally in the amount of morphine they contain.
Abbreviations
SC: standard concentration, WFI: sterile water for injection, Br: bromide, HCl: hydrochloride, Na: sodium, $x1H_2O$: hydrate

			☐ Not applicable<br>☐ Alternative SC with reason:
**Piperacillin/ Tazobactam**	80/ 10 mg/mL	short infusion, also prolonged	☐ Yes<br>☐ Not applicable<br>☐ Alternative SC with reason:
**Piritramide**	0.1 mg/mL<br>1 mg/mL	short infusion	☐ Yes<br>☐ Yes<br>☐ Not applicable<br>☐ Alternative SC with reason:
**Prednisolone-21-hydrogen-succinate**	1 mg/mL	bolus	☐ Yes<br>☐ Not applicable<br>☐ Alternative SC with reason:
**Propofol**	10 mg/mL	bolus	☐ Yes<br>☐ Not applicable<br>☐ Alternative SC with reason:
**Sildenafil citrate**	0.8 mg/mL<br>0.16 mg/mL	bolus/ short infusion<br>continuous infusion	☐ Yes<br>☐ Yes<br>☐ Not applicable<br>☐ Alternative SC with reason:
**Teicoplanin**	10 mg/mL	short infusion	☐ Yes<br>☐ Not applicable<br>☐ Alternative SC with reason:
**Theophylline**	1 mg/mL	continuous infusion	☐ Yes<br>☐ Not applicable<br>☐ Alternative SC with reason:
**Vancomycin HCl**	5 mg/mL	short infusion	☐ Yes<br>☐ Not applicable<br>☐ Alternative SC with reason:
**Vecuronium Br**	0.1 mg/mL	bolus/ continuous infusion	☐ Yes<br>☐ Not applicable<br>☐ Alternative SC with reason:

* For ceftazidime and cefuroxime, volume expansion occurs during reconstitution. The resulting concentration is slightly lower than the expected concentration. The concentrations listed here always refer to the actual concentration obtained during preparation.
** Manufacturers has been asked whether stability data for dilutions are available.
*** The hydrochloride and sulfate salts differ only minimally in the amount of morphine they contain.

Abbreviations

SC: standard concentration, WFI: sterile water for injection, Br: bromide, HCl: hydrochloride, Na: sodium, $x1H_2O$: hydrate
